# *Plasmodium yoelii* infection induces lung injury by modulating type 2 conventional dendritic cells autophagy via the STAT3-IRF4 signaling

**DOI:** 10.1038/s41419-026-08675-4

**Published:** 2026-04-10

**Authors:** Cansheng Hong, Guorong Deng, Zhihan Jiang, Yishuai Lu, Qianlian Wu, Chao Huang, Jia Tang, Haiqi Zhou, Qinan Liu, Xiujuan Luo, Yi Zhao, Yanwei Qi, Qingqing Li, Xiancai Ma, Quan Yang

**Affiliations:** 1 Department of Pathogenic Biology and Immunology, Sino-French Hoffmann Institute, School of Basic Medical Sciences, Guangzhou Medical University, Guangzhou, China; 2 Guangdong Provincial Key Laboratory of Allergy and Clinical Immunology, Guangzhou Medical University, Guangzhou, China; 3 State Key Laboratory of Respiratory Disease, National Clinical Research Center for Respiratory Disease, Guangzhou Institute of Respiratory Health, the First Affiliated Hospital of Guangzhou Medical University, Guangzhou, China; 4 Guangzhou National Laboratory, Guangzhou International Bio-Island, Guangzhou, China; 5 The Affiliated TCM Hospital of Guangzhou Medical University, Guangzhou, China

**Keywords:** Malaria, Malaria

## Abstract

Malaria is an infectious disease caused by *Plasmodium* that severely impacts human health, often resulting in lung injury. Classical type 2 dendritic cells (cDC2) in the lungs play a crucial role in the pathogenesis of asthma and infectious diseases; however, their specific functions during *Plasmodium* infection remain poorly understood. In this study, we demonstrated a significant accumulation and activation of cDC2 in the lungs of mice infected with *Plasmodium*. While the phagocytosis ability of activated cDC2 decreases, it promotes the differentiation of CD4^+^ T cells towards Th1 cells, thereby exacerbating lung injury. During our investigation into cDC2 accumulation in the lungs, we discovered that this accumulation may occur through autophagy. Furthermore, mechanistic studies revealed that the effect of *Plasmodium* on cDC2 is mediated by the JAK/STAT3 signaling pathway. Inhibition of STAT3 phosphorylation by the JAK inhibitor JSI-124 almost completely abolished the influence of *Plasmodium* on cDC2. The role of cDC2 in lung injury induced by *Plasmodium* infection was further substantiated in IRF4-deficient mice infected with *Plasmodium*. In conclusion, our research significantly enriches our understanding of lung cDC2, further elucidates the pathogenic mechanisms of *Plasmodium* infection, and offers a novel theoretical foundation for malaria prevention and control.

**Figure:**
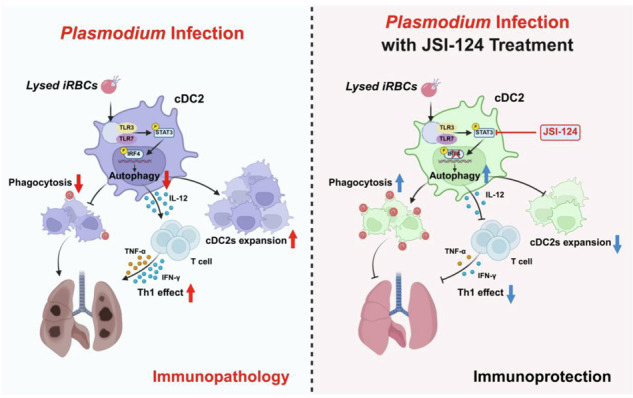
Schematic model demonstrated that *Plasmodium* infection suppressed autophagy in lung cDC2 via the TLR3/7-STAT3-IRF4 signaling pathway. Autophagy deficiency caused abnormal accumulation of cDC2 in lungs, impairing their phagocytosis capacity and clearance of malaria parasites. IL-12 secreted by cDC2 drove Th1-biased T-cell differentiation, which exacerbated pro-inflammatory responses and increased immunopathological damage in lung tissues. Inhibition of STAT3 or knockout of IRF4 within cDC2 restored cDC2 autophagy, reduced aberrant accumulation, re-established the phagocytic function of cDC2, and attenuated pro-inflammatory responses of Th1 cells, ultimately alleviating lung inflammatory damage.

Schematic model demonstrated that *Plasmodium* infection suppressed autophagy in lung cDC2 via the TLR3/7-STAT3-IRF4 signaling pathway. Autophagy deficiency caused abnormal accumulation of cDC2 in lungs, impairing their phagocytosis capacity and clearance of malaria parasites. IL-12 secreted by cDC2 drove Th1-biased T-cell differentiation, which exacerbated pro-inflammatory responses and increased immunopathological damage in lung tissues. Inhibition of STAT3 or knockout of IRF4 within cDC2 restored cDC2 autophagy, reduced aberrant accumulation, re-established the phagocytic function of cDC2, and attenuated pro-inflammatory responses of Th1 cells, ultimately alleviating lung inflammatory damage.

## Introduction

Malaria is a perilous disease caused by *Plasmodium* parasites, transmitted to humans via the bite of infected female *Anopheles* mosquitoes, representing a considerable risk to world health. Malaria, alongside tuberculosis and HIV/AIDS, is acknowledged as one of the three principal public health issues by the World Health Organization (WHO) [1]. According to the “World Malaria Report 2024”, in 2023, there were around 263 million instances of malaria and 597,000 fatalities attributed to the disease globally [2]. Malaria frequently results in pulmonary consequences, such as pulmonary edema. In certain instances, it may induce acute respiratory distress syndrome, leading to the fast emergence of dyspnea and advancement to respiratory failure [3]. Malaria-induced pulmonary damage has a significant fatality rate [4], and in the absence of adequate treatment, it poses a serious threat to the patient’s life.

To counteract immunological damage from pathogen infection, the body establishes an extensive lymphatic network around the alveolar wall, therefore preserving the homeostasis of the pulmonary immune microenvironment [5]. Within this complex environment, type 2 conventional dendritic cells (cDC2) have garnered increasing attention due to their distinctive location and functional adaptability. cDC2 are a subset of dendritic cells (DCs) originating from hematopoietic stem cells (HSCs) within the bone marrow. The differentiation of cDC2 is significantly reliant on the Notch signaling pathway, the activation of which can stimulate the expression of the transcription factor RBP-Jκ, subsequently enhancing the transcription of cDC2-specific genes such as CD11b and SIRPα in conjunction with IRF4 [6]. cDC2 has a crucial role in combating pulmonary infections. Previous research indicates that pulmonary cDC2 exhibit a greater propensity to uptake allergens in asthma and dust mite models than cDC1, and induce stronger Th2 and Th17 responses to dust mites [7]. In models of Gram-negative bacterial infection and *Chlamydia trachomatis* infection, there is an increased production of chemokines such as IL-12 and IL-6 to eliminate pathogens [8]. cDC2 exposed to *Plasmodium falciparum*-infected red blood cells (iRBCs) maintain their capacity to present antigens and activate T cells, polarizing them into the Th1 subset proficient in secreting IFN-γ [9].

Autophagy is a cellular process that degrades and recycles intracellular components. This activity is essential for preserving intracellular integrity, eliminating damaged organelles and proteins, and addressing stress circumstances such as nutritional deprivation [10]. Research indicates that autophagy can eliminate infections and enhance their clearance via heterophagic autophagy [11]. Consequently, autophagy serves as a key defense mechanism against pathogens. In *Plasmodium* infection models, the enhancement of autophagy by red pulp macrophages in the spleen is advantageous for the elimination of malaria parasites [12]. Research on autophagy in DCs has demonstrated that autophagy is involved in various facets of DCs maturation, antigen presentation, cytokine synthesis, and T cell activation [13]. Autophagy has been demonstrated to significantly contribute to pulmonary inflammation. The lack of *Atg5* in AT2 cells exacerbates bleomycin-induced lung damage. In APCs, the ablation of mTOR significantly reduces the frequency of IL-4^+^ Th2 cells, while the frequency of Th17 cells in the lungs and BAL increases [14, 15].

Signal transducer and activator of transcription 3 (STAT3) is a member of the STAT protein family, functioning in both signal transduction and transcriptional regulation downstream of Janus-associated kinase (JAK) and tyrosine kinase 2 (Tyk2) signaling pathways [16]. STAT3 primarily resides in the cytoplasm of cells in an inactive state. Upon activation, STAT3 triggers phosphorylation, homodimerization, nuclear translocation, and DNA binding, hence facilitating tumor proliferation, differentiation, apoptosis, cell transformation, invasion, angiogenesis, and immune evasion [17]. Growing evidence indicates that persistent STAT3 activation may disrupt the regulation of growth control and autophagy, thereby facilitating cell growth and survival [18]. STAT3 is also expressed in immune cells, including lymphocytes, macrophages, and DCs, where it plays a pivotal role in anti-tumor and anti-infection immunity by regulating immune cell differentiation and function [19, 20]. In *Leishmania* infection, the suppression of STAT3 expression in DCs diminishes the formation of cDC2 and prolonged Th2 responses, consequently enhancing protection against *Leishmania* infection in mice [21].

Interferon regulatory factor 4 (IRF4), a member of the IRF family, is distinct from other members due to its expression being confined to hematopoietic and adipocyte lineages, with its activation not being driven by interferon [22]. IRF4 is crucial in modulating adaptive immunity, facilitating the differentiation, affinity maturation, and functionality of macrophages, DCs, T cells, and B cells [23]. Additionally, IRF4 is pivotal in carcinogenesis and immunological function, with its dysregulation intimately linked to the onset of autoimmune disorders, lymphoma, multiple myeloma, and other malignancies [24, 25]. IRF4 is implicated in monocytes, plasma cell-like dendritic cells (pDCs), and cDCs, especially in the formation and functionality of cDC2 [26–28]. In schistosomiasis infection, IRF4-dependent cDC2 are essential for facilitating type 2 immune responses in the lungs. Inhibiting IRF4 in cDC2 via both PRR-dependent and PRR-independent signaling can diminish Th2 responses and enhance Th17 responses [27]. Recent investigations have revealed pulmonary inflammation in both human and mouse models of schistosomiasis caused by *Schistosoma mansoni*, emphasizing the essential role of cDC2 and their reliance on IRF4 for optimal development and function [29].

This study examined the attributes of cDC2 in the pulmonary tissue of C57BL/6 mice infected with *Plasmodium* and analyzed the function and possible regulatory pathways of STAT3 and IRF4 in lung injury induced by autophagy within cDC2. The findings offer novel theoretical insights into the immunological regulatory systems of malaria and present innovative concepts for the prevention and treatment of malaria and associated disorders.

## Materials and methods

### Mice

All C57BL/6 mice used in this study were housed under specific pathogen-free (SPF) conditions at the Laboratory Animal Center of Guangzhou Medical University (Guangzhou, China), starting from 6-8 weeks of age. IRF4 conditional (floxed) mutant mice (IRF4^flox/flox^; Stock No. 009380) and LysM-Cre mice (B6N. 129P2 (B6) Lyz2tm1 (cre) Ifo/J; Stock No 018956) were originally purchased from the Jackson Laboratory (Bar Harbor, ME, USA) and maintained with a C57B/L6 background. 6-8 weeks old female C57BL/6 mice were purchased from the Animal Experimental Center of Sun Yat-Sen University (Guangzhou, China). To acquire IRF4 cKO mice, LysM-Cre mice mated with IRF4^flox/flox^ mice, and cohorts were established by mating F1 IRF4^flox/+^; Cre+ mice to littermate IRF4^flox/+^; Cre- mice. No randomization and blinding were used in animal experiments.

### Animal experiments and compound treatment

*Plasmodium yoelii* 17XNL (*P. y* 17XNL) was purchased from Malaria Research and Reference Reagent Resource Centre (MR4). C57BL/6 mice were intraperitoneally inoculated with *P. y* 17XNL parasites. Thin blood smears were prepared from tail vein blood, and parasitemia was quantified via microscopic examination of Giemsa-stained slides by calculating the mean percentage of infected red blood cells (iRBCs) relative to total RBCs. When parasitemia exceeded 10%, blood was collected from the ocular plexus of infected mice. After centrifugation to remove serum, RBCs were resuspended in PBS and subjected to Percoll gradient centrifugation (50% and 60%) for iRBC isolation. Purified iRBCs were cryopreserved at −80 °C. For infection, C57BL/6 mice were intraperitoneally injected with 1 × 10^6^ iRBCs. All methods were performed in accordance with the relevant guidelines and regulations. All animal experiments were conducted in strict accordance with the experimental animal welfare security system as required by the Experimental Animal Ethics Committee of Guangzhou Medical University to minimize the suffering of mice. The INF + PBS group mice were injected with solvent (10% DMSO in PBS), and the INF + JSI-124 group mice were injected with JSI-124 (1 mg/kg) in 10% DMSO in PBS. Starting from the third day, inject once every three days, and the INF + Artemisinin group mice were injected with Artemisinin (40 mg/kg per day).

### Reagents and antibodies

The RPMI 1640 medium (C11875093), fetal bovine serum (FBS) (10100147 C), penicillin–streptomycin (15140122), CFSE (C34554), and CM-H2DCFDA (C6827) were obtained from Invitrogen (Grand Island, NY). PMA (P1585), ionomycin (I3909), Brefeldin A (B5936), DMSO (D2650), and cucurbitacin I hydrate (JSI-124) (GC13347) were purchased from Sigma-Aldrich (St. Louis, MO). Rapamycin (T1537), Simvastatin (T0687), 3-AT (T67498) and Bafilomycin A1 (T6740) were purchased from Targetmol (Boston, MA). Artemisinin (B20993) was purchased from Yuanye (Shanghai, China). NO detection reagent (ENZ-51013) was purchased from Enzo Life Sciences (Farmingdale, USA). PGN-BS (tlrl-pgnb3) was purchased from InvivoGen (San Diego, CA, USA). Poly(I: C) (HY-107202), LPS (HY-D1056), R848 (HY-13740), CU-CPT 4a (HY-108473), DPTA NONOate (HY-W507393), and Enpatoran (HY-134581) were purchased from MCE (New Jersey, USA). Recombinant murine GM-CSF (AF31503) and IL-4 (P07750) were obtained from PeproTech (OakPark, CA). Red blood cell (RBC) lysis buffer (C3702) was obtained from Beyotime Biotechnology (Shanghai, China). Annexin V-FITC/PI apoptosis kit (AP101) was obtained from Multi Sciences (Hangzhou, China). STAT3 Rabbit pAb (T55292), p-STAT3 Rabbit pAb (T56566), GAPDH Rabbit pAb (649201), and HRP-conjugated secondary Rabbit pAb (M21007) were purchased from Abmart (Shanghai, China). IRF4 Rabbit pAb (A0524), LC3B Rabbit mAb (A19665), and p62 Rabbit mAb (A19700) were purchased from ABclonal (Beijing, China). Fluorescein-conjugated anti-mouse Abs including CD11b-PE-Cy7 (Clone M1/70, 101216), CD11b-APC (M1/70, 101212), F4/80-PE (BM8, 123110), Gr-1-FITC (RB6-8C5, 108406), Ly6G-APC-cy7 (1A8, 127623), CD4-PerCP-Cy5.5 (GK1.5, 100434), CD8a-APC-Cy7 (53-6.7, 100713), IL-6-APC (MP5-20F3, 504507), GM-CSF-APC (MP1-22E9, 505413), IL-10-PE (JES5-16E3, 505007), IL-1α-PE (ALF-161, 503203), IL-17α-PE (TC11-18H10.1, 506904), CD11c-PerCP-Cy5.5 (N418, 117328), CD11c-PerCP-Cy7 (N418, 117318), MCH II-FITC (AF6-120.1, 116405), MCH II-PerCP-Cy5.5 (N418, 117327), B220-APC (RA3-6B2, 103211), CD103-PE (2E7, 121406), CD103-APC-Cy7 (2E7, 121432), CD80-PE (16-10A1, 104707), CD86-APC (GL-1, 105011), CD40-PE (3-23, 124609), IFN-γ-APC (XMG1.2, 505809), IL-12-PE (C15.6, 505203), IL-4-PE (11B11, 504103), IRF4-PE (IRF4.3E4, 646403), TNFα-APC-Cy7 (MP6-XT22, 506343), and purified anti-mouse CD3 (17A2, 100202)/CD28 (37.51, 122002) antibody were purchased from Biolegend (San Diego, CA), CD3e-PE (145-2C11, 553064), CD8a-APC (53-6.7, 553035), CD8a-FITC (53-6.7, 553031), p-STAT3-Brilliant Violet 421 (13A3-1, 651009), p-p65-PE (K10-895.12.50, 558423), and p-AKT-PE (M89-61, 560378) were purchased from BD Biosciences (San Jose, CA), eFluor450-FVD (65086314) was purchased from eBioscience (San Diego, CA).

### Cell preparation

Upon the euthanasia of mice, the spleen and lungs were carefully excised for further analysis. Lymphocytes in the lung and spleen were isolated using a blood cell lysis buffer following mechanical grinding. The lungs were rinsed with PBS, sectioned into small fragments, and incubated in a digestion solution containing DNase I and collagenase IV for 30 min at 37 °C prior to mechanical homogenization. The mixture was then filtered through a 74 µm filter, and red blood cells were eliminated using a blood cell lysis buffer. The lymphocytes from each tissue were ultimately suspended in RPMI 1640 medium.

### Magnetic bead sorting

Mouse Lung CD11c^+^ cells were sorted using CD11c MicroBeads UltraPure, mouse (130-125-835, Miltenyi Biotec, Germany). Resuspend the cell pellet in 400 µL of buffer per 10⁸ total cells. Add 100 µL of CD11c MicroBeads UltraPure per 10⁸ total cells. Mix well and incubate for 10 min in the dark in the refrigerator (2−8 °C). Wash cells by adding 10 mL of buffer per 10⁸ cells and centrifuge at 300 × *g* for 10 min. Aspirate supernatant completely. Resuspend up to 10⁸ cells in 500 µL of buffer. Before separation, the separation buffer was used to wash the MS column (Miltenyi Biotec, 130-042-201). Then, the antibody-conjugated cells were injected into the column and washed twice with the sorting buffer. Finally, the magnetic field was removed, and the target cells were collected.

### Cell culture

One million single-cell suspensions of the mouse lung were placed in 48-well plates and cultured in RPMI 1640 medium containing 10% FBS, 10 ng/ml GM-CSF, and 20 ng/mL IL-4 at a condition of 37 °C and 5% CO_2_, and cells were collected on day 3.

### Quantitative real-time PCR

These experiments were performed according to the manufacturer’s instructions. RNA was extracted with a SteadyPure Universal RNA Extraction kit Ⅱ (Accurate Biotechnology, China), and cDNA was synthesized with a HiScript Ⅲ reverse transcriptase kit (Vazyme, China). Real-time PCR amplification was carried out in the presence of 1 µL of cDNA template, 5 µL of SYBR master mixture (Vazyme, China), and target gene-specific primers (Table S1) in a CFX Connect Real-time PCR Detection System (Bio-Rad, CA). Amplification of *β-actin* mRNA was used as an internal control.

### Western blotting

After cell lysis, the protein concentration was determined using the BCA protein quantitative kit (DingGuo Biotechnology, China). Protein samples were separated in 10% SDS denaturing polyacrylamide gels, transferred to nitrocellulose membranes, and subsequently blocked in 5% BSA for 1.5 h at room temperature. Specific primary Abs and HRP-conjugated secondary Abs were used to probe the target protein. Finally, the HRP chemiluminescent substrate kit (WBKLS0100, Millipore, MA) was used to detect signals of target proteins.

### Cell surface staining and Annexin V/PI staining

The cells were washed twice with PBS and then stained with specific fluorescence-labeled Abs for 30 min in the dark at 4 °C. These Abs targeted CD11c, MHC-II, CD11b, and CD103, respectively. For Annexin Ⅴ/PI staining, cells were washed with PBS after surface staining, followed by Annexin Ⅴ/PI staining according to the manufacturer’s instructions. The stained cells were analyzed by flow cytometry (FCM) (Beckman Coulter), and the results were analyzed utilizing CytoExpert 2.3 software (Beckman Coulter Fullerton, CA).

### Intracellular staining

For cytokine staining, cells were stimulated with 20 ng/ml PMA and 1 µg/ml ionomycin for 1 h at 37 °C under 5% CO_2_ conditions, then Brefeldin A was added for 4 h. After incubation, cells were washed with PBS, fixed with fixation and permeabilization solution (BD Biosciences) for 20 min at 4 °C in the dark, and permeabilized overnight at 4 °C in PBS buffer containing 0.1% saponin (Sigma), 1% BSA, and 0.05% NaN_3_. For intranuclear staining, cells were washed after surface staining, then fixed and permeated with nuclear-breaking solution, stained with specific fluorescence-labeled Abs (including p-STAT3, p-p65, IRF4, and p-AKT) for 30 min at 4 °C in the dark. The stained cells were analyzed by FCM, and the results were analyzed by CytoExpert 2.3 software.

### ROS quantification

Levels of produced reactive oxygen species (ROS) were measured with the oxidation-sensitive dye CM-H2DCFDA. Cells were incubated in RPMI 1640 medium at 37 °C for 30 min in the presence of 2 µM CM-H2DCFDA, and then labeled with CD11c, MHC-II and CD11b Abs at 4 °C in the dark. The levels of ROS in cells were quantified using FCM.

### NO quantification

About 0.5 million cells were resuspended in 2 mL of PBS, followed by centrifuging to remove supernatant. RPMI 1640 medium-diluted nitric oxide (NO) detection reagent was added to each tube at a ratio of 1:400. After mixing thoroughly, incubate the tube at 37 °C in the dark for 2 h, shaking every 30 min during the incubation process. Add 500 μL of matching cleaning solution to resuspend cells, followed by centrifuging to discard the supernatant. Subsequently, perform cell surface staining and FCM quantification.

### T cell proliferation assay

Splenocytes from normal mice were isolated and labeled with CFSE (5 μM, Invitrogen), stimulated with CD3/CD28 (1 μg/ml, Invitrogen) antibodies, and then cultured alone or cocultured with induced or sorted DCs at different ratios for 3 days. T cell proliferation was analyzed by FCM.

### Histology studies

After the mice were euthanized, the thorax and trachea were treated quickly to inject PBS and ensure adequate perfusion of the lungs, then fixed with 4% paraformaldehyde and embedded in paraffin and sliced. The sections were stained with conventional hematoxylin-eosin (H&E) and observed under a light microscope. Histological images were analyzed by Image J (National Institutes of Health, Bethesda, MD) software.

### Immunostaining

Paraffin sections of mouse lungs were initially prepared, deparaffinized, and rehydrated. Subsequently, high-pressure antigen retrieval was performed using sodium citrate solution. After washing, circles were drawn around the tissue with a hydrophobic barrier pen to fully enclose the tissue, followed by gentle absorption of residual moisture. Goat serum was then applied dropwise, and the container was sealed and maintained at room temperature for 1 h. Sections were then washed three times with PBS. Primary antibodies (against CD11b and CD11c, respectively) were diluted at 1:100 and applied to the sections, which were incubated overnight at 4 °C. Following primary incubation, slides were washed three times with PBS. Fluorescent secondary antibodies were dispensed dropwise to ensure complete tissue coverage, and slides were incubated in a light-proof chamber at room temperature for 1 h. Post-incubation washes were performed three times with PBS. Nuclei were counterstained with DAPI. Finally, imaging was conducted using a Leica fluorescence microscope.

As for cellular immunofluorescence, slides were inserted into a 24-well plate, followed by the uniform distribution of 5 × 10⁵ sorted lung CD11c⁺ cells onto slides for a 2-h culture period (In the phagocytosis assay, CFSE-labeled iRBCs were added to the co-culture and incubated for 5 h.), the culture medium was then removed, and washing was performed with PBS. Fixation was carried out with 4% paraformaldehyde. After washing with PBS, the blocking solution (10% WBR, 0.3% Triton X-100 in PBS) was added and left standing at room temperature for 1 h. Primary antibody was diluted 1:500 in diluent (PBS with 10% WBR and 0.3% Triton X-100) and added to the slides, followed by incubation at 4 °C overnight. Washing was performed with PBS containing 0.3% Triton X-100. Secondary antibody diluent was then added and incubated at room temperature for 1 h. After washing with PBS containing 0.3% Triton X-100, anti-quenching mounting medium was applied to a glass slide. The slide was inverted onto the coverslip and encircled with nail polish for stabilization. Image data were collected using a Zeiss confocal microscope.

### Statistical analysis

Statistical analysis was performed using unpaired *t*-test, one-way ANOVA, and two-way ANOVA. GraphPad Prism software (v8.01) was used for statistical analysis. A *P*-value of >0.05 was considered not statistically significant. A *P*-value of <0.05 was considered to be statistically significant and represented as an asterisk (*). A *P*-value of < 0.01 was considered to be more statistically significant and represented as double asterisks (**). A *P*-value of <0.001 was considered to be the most statistically significant and represented as triple asterisks (***).

## Results

### Plasmodium infection resulted in pulmonary injury and accumulation of cDC2 in the mouse lung

**Fig. 1:**
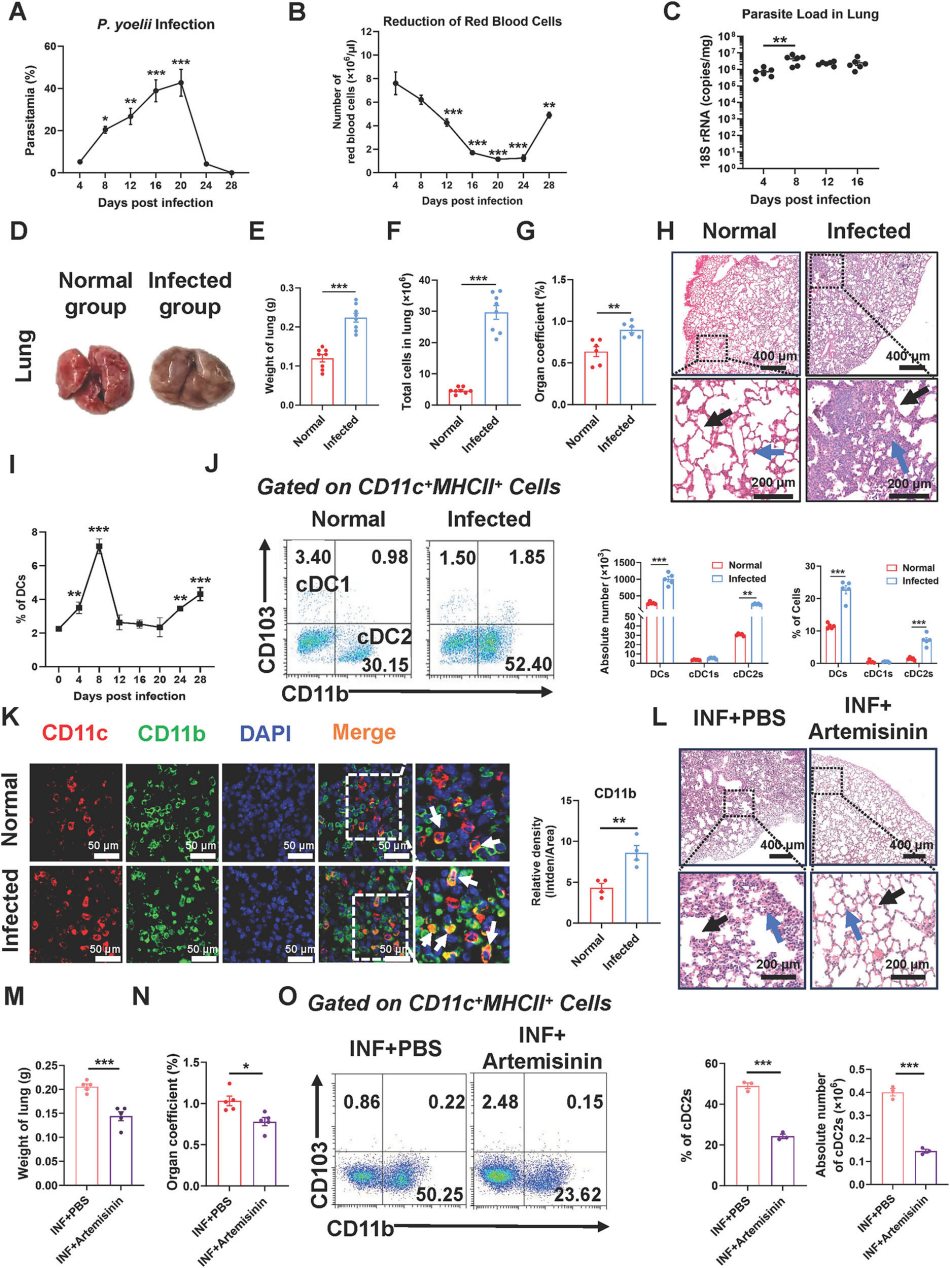
*Plasmodium* infection resulted in pulmonary injury and cDC2 accumulation. C57BL/6 mice were intraperitoneally injected with 1 × 10^6^ infected red blood cells. **A**, **B** The parasitemia erythrocyte counts were dynamically monitored from 0 to 28 dpi (*n* = 4). Dynamic monitoring of *Plasmodium*-specific gene 18S rRNA expression in lungs from 4 to 16 dpi (**C**). Representative images of lung (**D**), weight of lung (**E**), total cells in lung (**F**) and organ coefficient (**G**) from normal and infected mice. Representative images of H&E staining of lungs: Black arrows represent alveolar morphology, and blue arrows represent leukocyte infiltration. Scale bar: 400 μm and 200 μm (**H**). Dynamic monitoring of the proportion of DCs in lungs via FCM from 0 to 28 dpi (**I**). FCM was used to detect and analysis the proportion and absolute number of CD11c^+^ DCs, CD11c^+^MHC-II^+^CD11b^+^ cells (cDC2), and CD11c^+^MHC-II^+^CD103^+^ cells (cDC1) on the 8 dpi (*n* = 5) (**J**). Representative immunofluorescence images of lung CD11c^+^CD11b^+^ cells (**K**). Representative images of H&E staining of lungs from INF + PBS and INF + Artemisinin group (**L**). Statistical graph of lung weight (**M**) and organ coefficient (**N**) from two groups. The proportion and absolute number of cDC2 from two groups (**O**). The data comes from individual mice in independent experiments. The data is expressed as mean ± standard deviation, *,*P* < 0.05；**,*P* < 0.01；***, *P* < 0.001.

To evaluate the impact of *Plasmodium* infection on the lungs and pulmonary cDC2 in mice, we developed a *Plasmodium* infection model utilizing C57BL/6 mice infected with *Plasmodium yoelii* 17XNL (*P.y* 17XNL). Mice received intraperitoneal injections of *P.y* 17XNL-infected red blood cells (iRBCs). Peripheral blood was obtained on day 4 post-infection, stained with Giemsa, and analyzed to assess the infection state. The findings indicated a gradual rise in parasitemia, reaching its peak on day 20 post-infection (Fig. 1A). The total clearance of infection was achieved on day 28. Conversely, the peripheral red blood cell (RBC) counts diminished throughout the illness, with a slow recovery commencing after day 20 (Fig. 1B). During the infection, mice exhibited diminished activity, clustering together in the cage, with some having unkempt fur and body inversion. In contrast, uninfected mice had increased activity and possessed silky, lustrous coats. Moreover, we quantified the expression of *Plasmodium*-specific 18S rRNA gene in lung tissues from mice at different post-infection days using qPCR. The results showed that the expression levels peaked on day 8 post-infection and were significantly different compared to those at 4 days post-infection (*P* < 0.01) (Fig. 1C).

Subsequently, 6-8-week-old SPF-grade C57BL/6 mice were randomly allocated to an infected group and a normal group. Mice from both groups were euthanized on day 8 post-infection, and their lungs were harvested. Substantial pathological alterations were noted in the lungs of the infected mice. The lung had a pallid appearance with a darker texture (Fig. 1D). In comparison to normal mice, the infected mice demonstrated a considerable increase in lung weight (*P* < 0.001), total lung cell counts (*P* < 0.001), and organ coefficient (*P* < 0.01), demonstrating significant edema, exudation, and other pathological changes in the lung (Fig. 1E–G). Furthermore, paraffin slices of lung from both groups were produced for hematoxylin and eosin (H&E) staining. The infected group displayed phenotypic alterations, including alveolar septal thickening, structural disorganization, and lymphocyte infiltration, indicating pulmonary parenchyma damage due to *Plasmodium* infection (Fig. 1H).

Considering the crucial function of DCs in the immunological regulation within the lung, we sought to examine the distribution of DCs and their subsets within the lungs of mice. First, we dynamically monitored the changes in DCs within the lungs of mice at different post-infection days using FCM. The results showed that the proportion of DCs peaked at day 8 post-infection (Figs. 1I and S1A). Based on this time point, we proceeded to investigate the changes in DC subsets before and after infection. FCM analysis revealed that, in comparison to mice from the normal group, mice from the infected group exhibited a significant increase in both the proportion (*P* < 0.01) and absolute number (*P* < 0.001) of cDC2 in the lungs (Figs. 1J and S1B). We subsequently performed immunofluorescence experiments on lung from both normal and infected mice, utilizing fluorescence-conjugated primary antibodies against CD11c and CD11b to label cDC2. The findings revealed that cDC2 were present in the lungs of both normal and infected mice, while a distinct aggregation of cDC2 was observed in the lungs of infected mice; the relative intensity of CD11b was higher in the infected group (*P* < 0.01) than in the normal group (Fig. 1K). The findings indicated that *Plasmodium* infection not only induced significant pathological damage to the lungs of mice but also promoted local enrichment of cDC2 by altering the distribution of DC subsets within the lungs of mice. In addition, we also observed that the proportions of myeloid-derived suppressor cells (MDSCs) and neutrophils in the lungs of infected mice were significantly higher than those in the normal group, suggesting that lung injury induced by *Plasmodium* may be a collective outcome caused by the synergistic effects of multiple cell types (Fig. S1C). To evaluate whether anti-*Plasmodium* treatment could ameliorate lung function in infected mice, we administered artemisinin to the infected mice. H&E staining results demonstrated that artemisinin treatment alleviated inflammation (Fig. 1L), preserved alveolar structure integrity, and improved both lung weight (*P* < 0.001) and lung coefficient (*P* < 0.05) in the mice (Fig. 1M, N). Furthermore, under the effect of artemisinin, the proportion (*P* < 0.001) and absolute number (*P* < 0.001) of cDC2 in the lungs were significantly reduced (Fig. 1O), indicating that anti-malarial therapy effectively ameliorates lung function.

### Plasmodium infection promoted the proliferation and activation of cDC2 in the lungs of mice through iRBCs

**Fig. 2:**
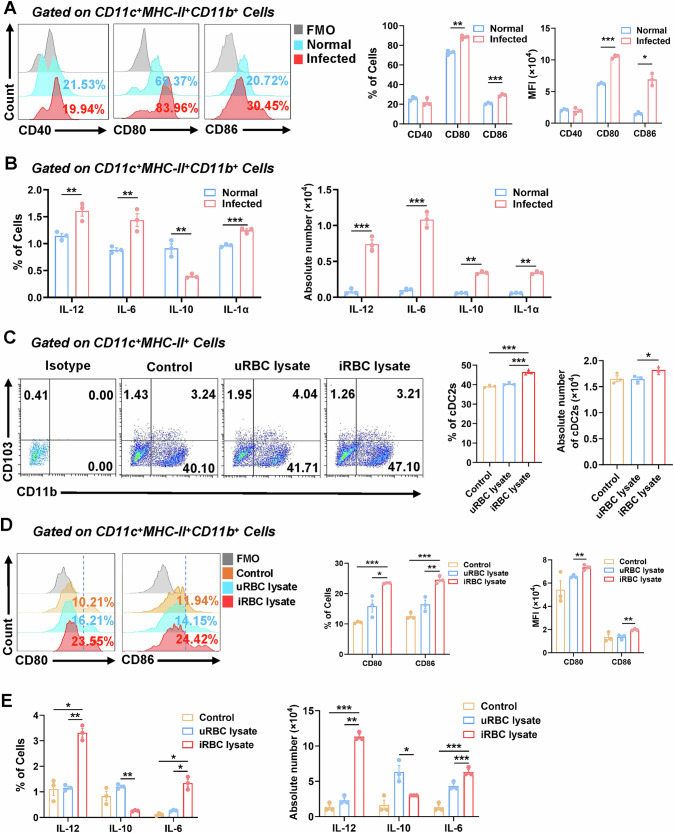
*Plasmodium* infection promoted the proliferation and activation of cDC2 via iRBCs. Normal and malaria-infected mice were euthanized on the 8 dpi, the activation of lung cDC2 (**A**) and cytokine secretion (**B**) were detected by FCM using lung single-cell suspension. The relevant components of *Plasmodium* (iRBC lysate) were used to treat lung cells in vitro. After co-culturing for 3 days, the proportion (**C**), activation (**D**), and cytokine secretion (**E**) of pulmonary cDC2 were detected by FCM. Date was presented as the mean ± SD and representative of three independent experiments. *, *P* < 0.05；**, *P* < 0.01；***, *P* < 0.001.

cDC2 provides a pivotal function as mediators between innate and adaptive immune responses, especially during pathogen invasion in the pulmonary system of mice [7]. Specifically, cDC2 can elicit localized immunological responses through T cell activation. To examine the function of cDC2 in the lungs of infected mice, we harvested lungs from both normal and infected mice and generated single-cell suspensions. The expression levels of activation molecules, including CD40, CD80, and CD86, on cDC2 were subsequently assessed using FCM. The findings indicated that the proportion (*P* < 0.01) and mean fluorescence intensity (MFI) (*P* < 0.001) of CD80 on cDC2 in lungs of infected mice were markedly elevated compared to the normal group (Fig. 2A). The proportion (*P* < 0.001) and MFI (*P* < 0.05) of CD86 on cDC2 in the infected mice were also considerably increased relative to the normal, demonstrating that *Plasmodium* infection markedly promoted cDC2 activation within mouse lungs.

Additionally, cDC2 in the lungs of infected mice contributed to immune regulation through the secretion of diverse cytokines. FCM examination demonstrated that cDC2 in lungs of infected mice displayed markedly elevated secretion of IL-12 (*P* < 0.01), IL-6 (*P* < 0.01) and IL-1α (*P* < 0.001) compared to the normal, whereas IL-10 secretion was dramatically diminished (*P* < 0.01) in the infected mice. Upon quantifying absolute cytokine production, cDC2 from lungs of infected mice also exhibited significantly elevated levels of IL-12 (*P* < 0.001), IL-6 (*P* < 0.001) and IL-1α (*P* < 0.01) relative to the normal group (Figs. 2B and S1D). We noticed that the absolute numbers of IL-10-positive cDC2 were also significantly upregulated in the lungs of infected mice (*P* < 0.01), which can be attributed to the overall expansion of cell populations in the lungs of infected mice. The gene expression data consistently demonstrated that DCs in the lungs of infected mice exhibited elevated levels of IL-12 (Fig. S1E), implying that cDC2 in the lungs may influence T cell differentiation by facilitating Th1-type cytokine production.

The activation of cDC2 in lungs is modulated by several variables, including inflammatory cytokines and chemokines, which engage cDC2 via multiple routes in pathological circumstances. To clarify the mechanism by which *Plasmodium* infection enhances cDC2 activation in the lungs, we evaluated the impact of *Plasmodium* components, particularly iRBCs, on cDC2 in the lungs via in vitro tests. The findings indicated that the percentage of cDC2 in lungs of mice administered iRBC lysate for 3 days was markedly elevated compared to the group receiving uninfected red blood cell lysate (uRBC lysate) (*P* < 0.001) (Fig. 2C). Furthermore, the proportion and MFI of cDC2 exhibiting activation markers CD80 (*P* < 0.05) and CD86 (*P* < 0.01), along with their overall activation (*P* < 0.01), were markedly elevated in the iRBC lysate-treated group relative to the uRBC lysate-treated group (Fig. 2D). Moreover, cDC2 in the lungs of mice treated with iRBC lysate exhibited markedly elevated levels of IL-12 (*P* < 0.01) in comparison to the uRBC lysate-treated group (Figs. 2E and S1F). In summary, *Plasmodium* infection enhanced the proliferation and activation of cDC2 in the pulmonary region of mice via iRBCs.

### Pulmonary cDC2 promoted T cell differentiation towards Th1 after Plasmodium infection

**Fig. 3:**
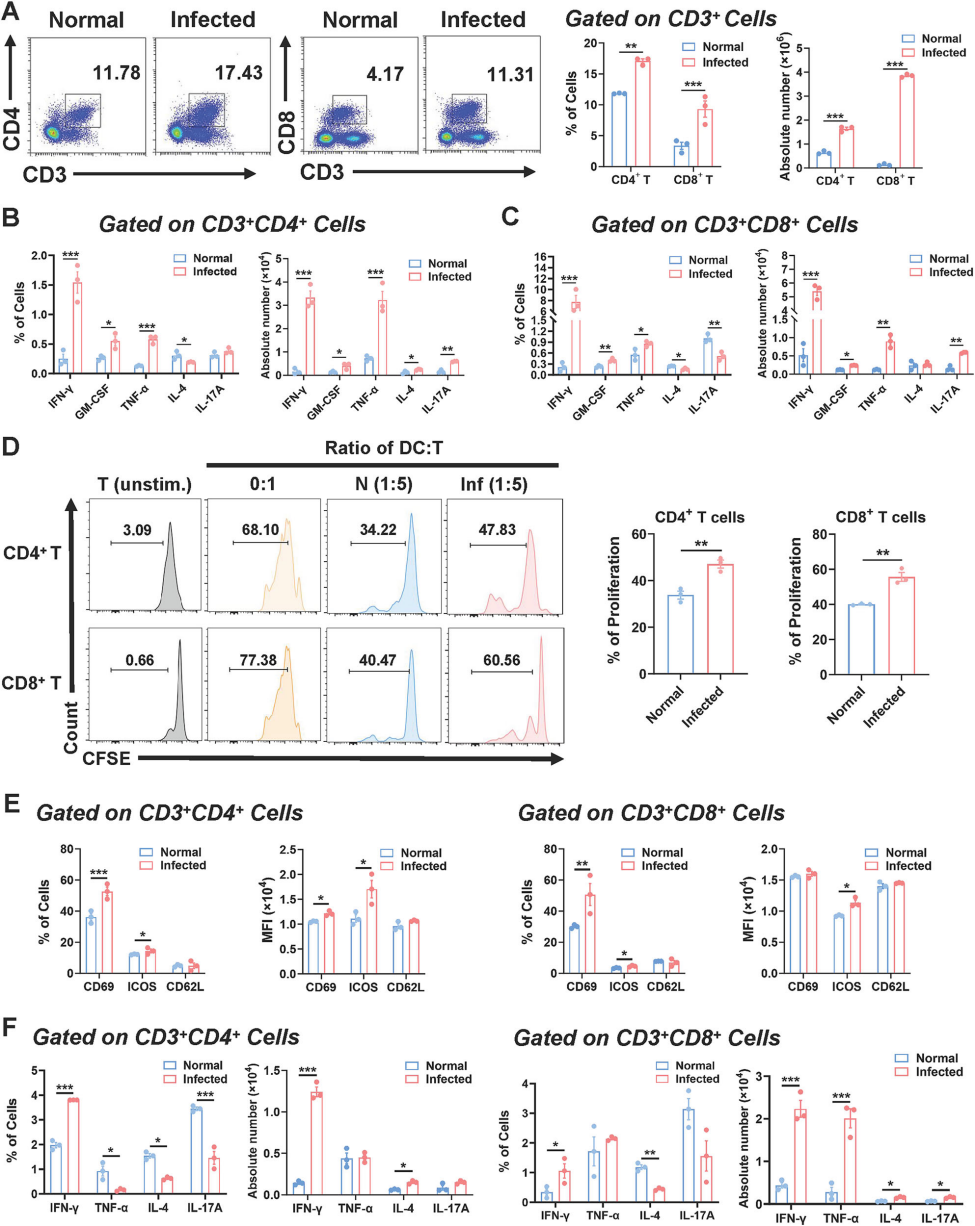
Pulmonary cDC2 promoted Th1-biased T cell differentiation upon *Plasmodium* infection. C57BL/6 mice were infected with *P. yoelii* and sacrificed 8 dpi., the proportion and the absolute number of CD4^+^ T and CD8^+^ T cells (**A**) as well as cytokine secretion (**B**, **C**) were detected by flow cytometry using lung single-cell suspension. Then, normal and infected mice lung CD11c^+^ DCs were sorted and co-cultured with T cells. After 3 days, T cell proliferation (**D**), activation (**E**) and cytokine secretion (**F**) were detected by FCM. Each group consists of 3-5 mice, and each experiment is repeated 2-3 times. Using an unpaired two tailed t-test, *, *P* < 0.05；**, *P* < 0.01；***, *P* < 0.001.

DCs and their subsets serve a crucial function as antigen-presenting cells in mice, with their activities manifesting as alterations in T cell phenotypes. To clarify the immunological regulatory function of cDC2 in lungs during *Plasmodium* infection, we examined the development and activity of T cells in lungs of both normal and infected mice. We first analyzed percentages of CD4^+^ T and CD8^+^ T cells in the lung. FCM demonstrated that the proportion (*P* < 0.01) and absolute number (*P* < 0.001) of CD4^+^ T cells in the lungs of infected mice were markedly elevated compared to the normal group. Similarly, the proportion (*P* < 0.001) and absolute number (*P* < 0.001) of CD8^+^ T cells in the lungs of infected mice were also significantly increased compared to the normal group (Fig. 3A). The data indicated that *Plasmodium* infection drove T cell migration to mouse lungs.

Upon noting that *Plasmodium* infection prompted T cell increase in lungs, we conducted further investigation into the function of these T cells. FCM analysis demonstrated that the percentage of CD4^+^ T cells producing IFN-γ (*P* < 0.001), GM-CSF (*P* < 0.05), and TNF-α (*P* < 0.001) in lungs of infected mice was markedly elevated compared to normal mice (Figs. 3B and S2A). Moreover, the absolute number of CD4^+^ T cells producing IFN-γ (*P* < 0.001), GM-CSF (*P* < 0.05), TNF-α (*P* < 0.001) and IL-17A (*P* < 0.01) was markedly increased in infected mice. In contrast, the percentage of CD4^+^ T cells producing IL-4 was markedly reduced in infected mice relative to normal mice (*P* < 0.05), indicating that *Plasmodium* infection promotes CD4^+^ T cell differentiation towards the Th1 phenotype. We additionally evaluated the cytokine secretion profile of CD8^+^ T cells. The percentage of CD8^+^ T cells producing IFN-γ (*P* < 0.001), GM-CSF (*P* < 0.01), and TNF-α (*P* < 0.05) in lungs of infected mice was also markedly elevated compared to that in lungs of normal mice (Figs. 3C and S2A). The absolute numbers of CD8^+^ T cells producing IFN-γ (*P* < 0.001), GM-CSF (*P* < 0.05), and TNF-α (*P* < 0.01) were considerably elevated in infected mice. The expression of IL-17A was markedly reduced in the lungs of infected mice (*P* < 0.01) relative to controls, indicating increased cytotoxic activity of CD8^+^ T cells in infected lungs. In conclusion, our findings demonstrated that *Plasmodium* infection facilitated the development of CD4^+^ T cells into the Th1 type and amplified the cytotoxic capabilities of CD8^+^ T cells through the induction of IL-12 release from cDC2, thereby reinforcing the anti-malarial immune response. However, overactivated T cells may induce immune-mediated damage in lung tissues through pro-inflammatory molecules such as IFN-γ and TNF-α.

We further investigated the ability of *Plasmodium* infection to induce lung DCs in mice to promote T cell responses. We sorted CD11c^+^ DCs from the lungs of control and infected mice using magnetic beads and co-cultured them with CFSE-labeled normal mouse splenocytes. The proliferation of T cells was then detected by FCM. The results showed that compared to normal mice, isolated lung CD11c^+^ DCs from infected mice significantly promoted the proliferation of CD4^+^ T (*P* < 0.01) and CD8^+^ T (*P* < 0.01) cells (Fig. 3D). Meanwhile, the proportion of early activation marker CD69 in CD4^+^ T (*P* < 0.001) and CD8^+^ T (*P* < 0.01) cells in infected mice was significantly higher than that in normal mice (Figs. 3E and S2B). The proportion of ICOS (CD4^+^ T, *P* < 0.05; CD8^+^ T, *P* < 0.05) and the MFI of ICOS (CD4^+^ T, *P* < 0.05; CD8^+^ T, *P* < 0.05) in infected mice were also significantly higher than those in normal mice. However, the proportion and MFI of CD62L on both CD4^+^ and CD8^+^ T cells in infected mice showed no significant difference compared to normal mice (Fig. 3E). We conducted a more in-depth analysis of cytokine production by T cells. The findings indicated that IFN-γ secretion in CD4^+^ T cells from infected mice was markedly elevated compared to normal mice (*P* < 0.001), and the absolute number of IFN-γ-secreting CD4^+^ T cells was also dramatically increased (*P* < 0.001) (Figs. 3F and S2C). However, levels of TNF-α (*P* < 0.05), IL-4 (*P* < 0.05), and IL-17A (*P* < 0.001) produced by CD4^+^ T cells from infected mice were markedly reduced compared to those of normal mice. In CD8^+^ T cells, IFN-γ secretion was also markedly elevated in infected mice relative to normal mice (*P* < 0.05), whereas IL-4 secretion was decreased (*P* < 0.01). No significant alterations were seen in the secretion of TNF-α or IL-17A between infected and normal mice. These findings demonstrated that *Plasmodium* infection stimulated T cell proliferation and functionality via a cDC2-dependent pathway in mouse lungs, influencing both the immune response and pathological processes.

### Plasmodium infection inhibited the phagocytic function of cDC2 in the lungs of mice

**Fig. 4:**
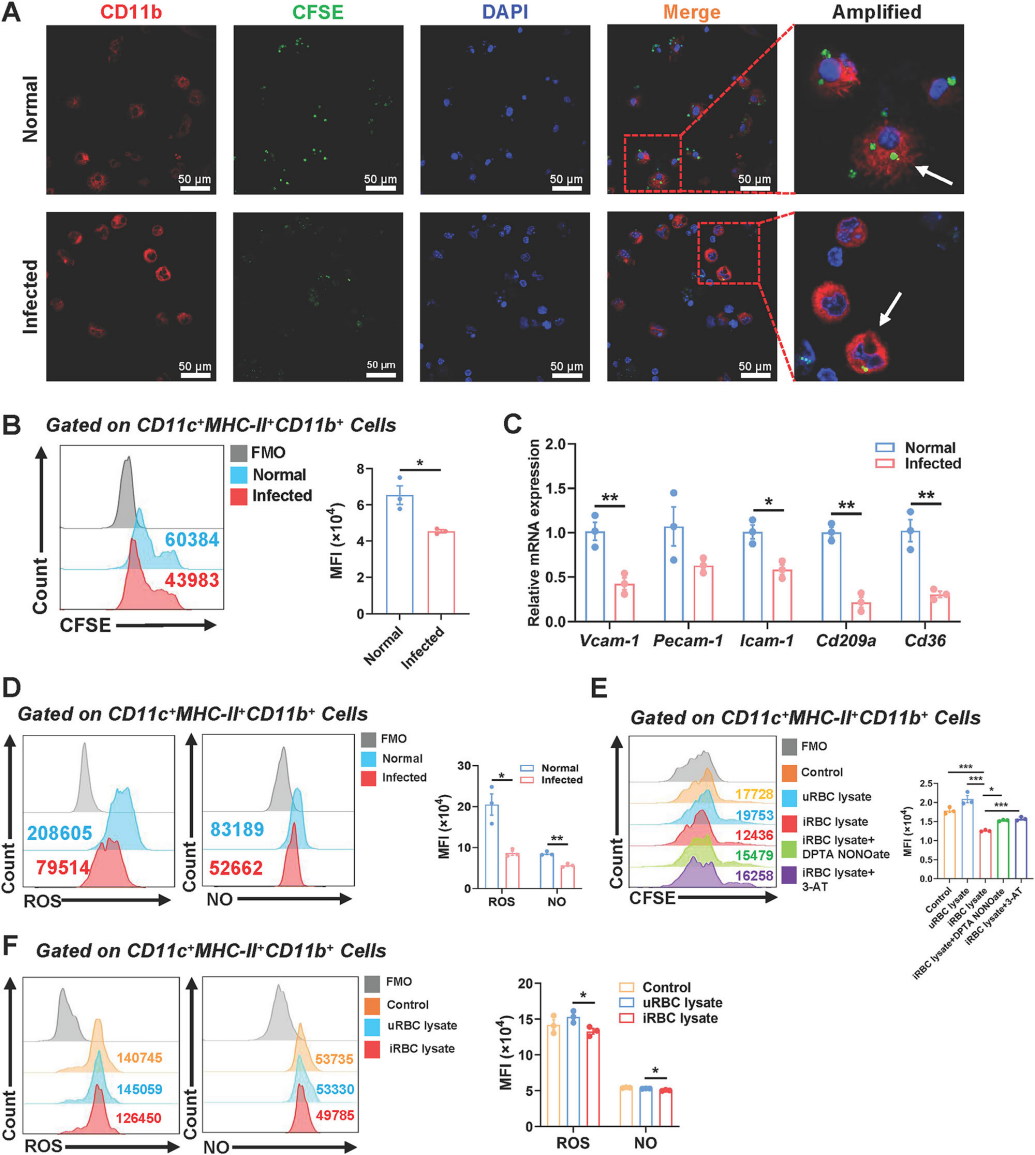
*Plasmodium* infection inhibited the phagocytic function of cDC2. iRBCs were stained with CFSE and co-cultured with sorted pulmonary CD11c^+^ DCs from normal and infected mice for 5 h. Confocal microscopy images of CD11b (red) and nucleus staining (blue) in pulmonary cDC2, iRBCs-CFSE (green). Scale bars: 50 μm(**A**). After co-culturing lung cells with iRBCs-CFSE, the average fluorescence intensity of CFSE in pulmonary cDC2 was detected by FCM (**B**). Pulmonary CD11c^+^ DCs gene expression from sorted normal and infected mice (**C**). ROS and NO levels were measured from normal and infected mice by FCM (**D**). uRBC lysate, iRBC lysate, DPTA NONOate (5 μM), and 3-AT (6 mM) were used to treat lung cells. After co-culturing, the average fluorescence intensity of iRBCs-CFSE (**E**), ROS and NO levels in pulmonary cDC2 were detected by FCM (**F**). Each group consists of 3-5 mice, and each experiment is repeated 2–3 times. Using an unpaired two tailed t-test, *, *P* < 0.05；**, *P* < 0.01；***, *P* < 0.001.

DCs, akin to macrophages and neutrophils, are specialized phagocytes proficient in internalizing antigens. To assess the impact of *Plasmodium* infection on the phagocytic ability of pulmonary cDC2, we investigated their functionality both in vivo and in vitro. CD11c⁺ DCs were isolated from the lungs of both normal and infected mice and subsequently co-cultured with CFSE-labeled iRBCs. Immunofluorescence examination demonstrated that cDC2 from infected mice phagocytosed markedly fewer iRBCs compared to normal mice (Fig. 4A). FCM analysis of lung cell suspensions incubated with CFSE-labeled iRBCs confirmed this observation. The MFI of ingested iRBCs in cDC2 from infected mice was significantly lower than that in normal mice (*P* < 0.05) (Fig. 4B).

To investigate the mechanism behind this impairment, we measured mRNA expression levels of critical phagocytosis-related adhesion molecules in pulmonary CD11c⁺ DCs. Transcripts for *Vcam1, Icam1, Cd209a*, and *Cd36* were all markedly down-regulated in infected mice (Fig. 4C). Given that reactive oxygen species (ROS) and nitric oxide (NO) are essential effector molecules for phagocytic destruction, we subsequently assessed their intracellular concentrations within cDC2. The MFI of ROS (*P* < 0.05) and NO (*P* < 0.01) in pulmonary cDC2 from infected mice was dramatically diminished compared to controls (Fig. 4D). Compared with the uRBC lysate group, exposure of Pulmonary cDC2 cells to iRBC lysate resulted in a significant reduction in iRBC uptake capacity (*P* < 0.001). To further investigate the association between the decreased phagocytic capacity and the reduced ROS/NO levels, we treated the cells with the NO donor, DPTA NONOate and catalase inhibitor 3-AT. The results showed that treatment with DPTA NONOate group (*P* < 0.05) and 3-AT group (*P* < 0.001) effectively restored the ability of cDC2 to phagocytose iRBCs (Fig. 4E). Furthermore, compared with the uRBC lysate group, the generation of ROS (*P* < 0.05) and NO (*P* < 0.05) was also significantly decreased in cells treated with iRBC lysate (Fig. 4F).

### Plasmodium infection impeded the autophagy of cDC2 in mice lungs by stimulating the STAT3 signaling pathway

**Fig. 5:**
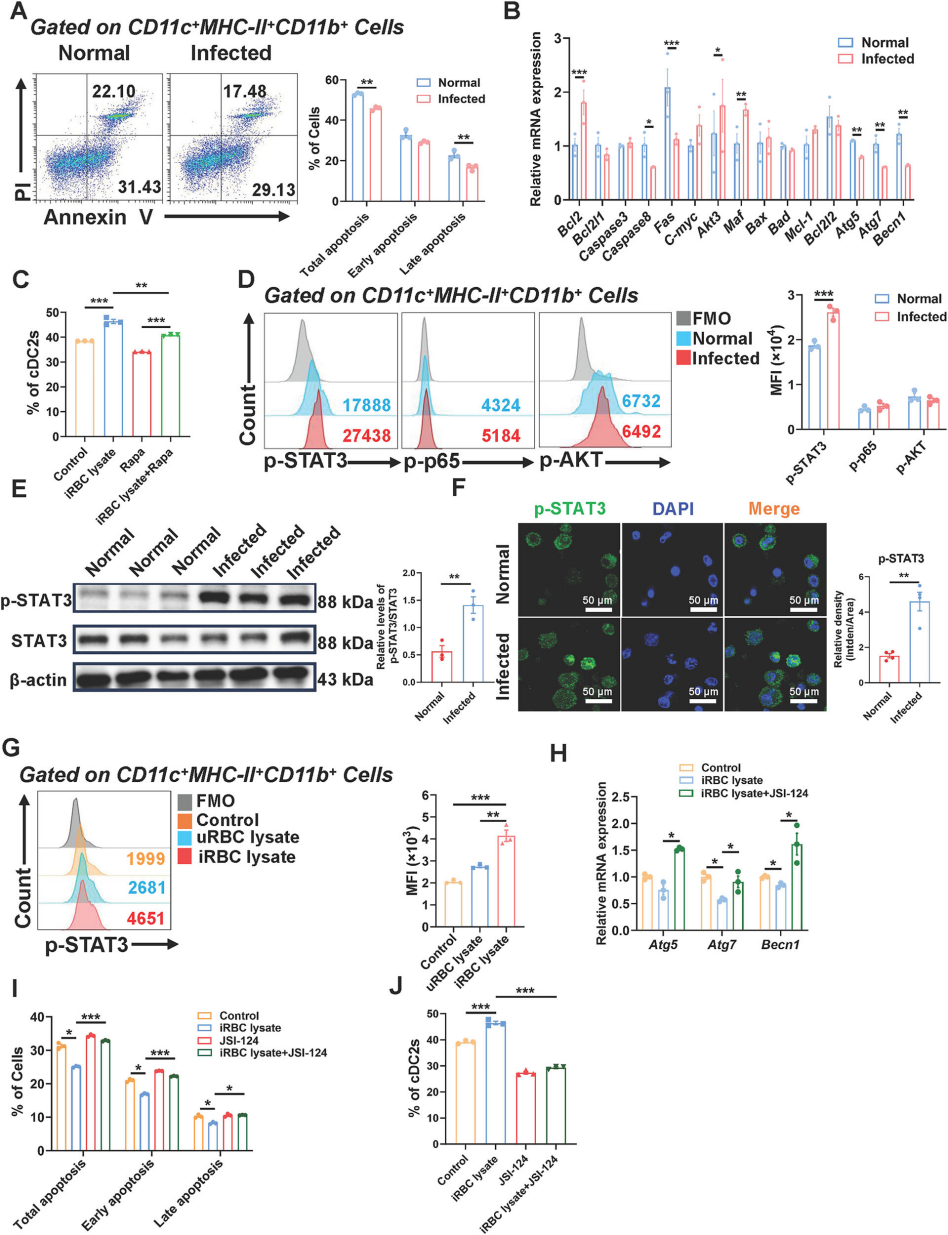
*Plasmodium* infection prevented cDC2 autophagy via activating STAT3. The apoptosis of pulmonary cDC2 from normal and infected mice was detected by FCM (**A**). RT-qPCR detection of pulmonary CD11c^+^ DCs genes related to apoptosis and autophagy (**B**). The lung cells of normal mice were cultured with GM-CSF (10 ng/mL), IL-4 (20 ng/mL), RBCs lysates (10million/mL) and Rapamycin(5 nM) for 3 days, the percentage of cDC2 were detected by FCM (**C**), the level of p-STAT3, p-p65 and p-AKT of pulmonary cDC2 from normal and infected mice were detected by FCM (**D**). The protein levels of STAT3 and p-STAT3 were detected by Western blotting on CD11c^+^ cells isolated from normal and infected mice (**E**). Confocal microscopy images of p-STAT3 (green) and nucleus staining (blue) from sorted pulmonary CD11c^+^ DCs. Scale bars: 50 μm (**F**). In vitro culture system, RBCs lysates were used to treat lung cells with, and the phosphorylation level of STAT3 was detected by FCM (**G**). The lung cells of normal mice were cultured with RBCs lysates and JSI-124 (500 nM) for 3 days. RT-qPCR detection of lung cells genes related to autophagy (**H**). The apoptosis of cDC2 was detected by FCM (**I**). The percentage of cDC2 was detected by FCM (**J**). Each group consists of 3–5 mice, and each experiment is repeated 2–3 times. Using an unpaired two tailed t-test, *, *P* < 0.05；**, *P* < 0.01；***, *P* < 0.001.

Apoptosis is a significant predictor of DCs lifetime, and fast apoptosis is the body's defense mechanism. On one hand, abnormalities in DCs apoptosis cause a buildup of DCs [30]. On the other hand, the rapid death of infected DCs can prevent a significant number of pathogens from entering the extracellular space and being phagocytosed by phagocytes [31]. However, the relationship between apoptosis and cDC2 is uncertain. To explore the molecular pathways by which *Plasmodium* infection prompts the proliferation of cDC2 in the lung of mice, we assessed the impact of infection on cDC2 apoptosis. We employed FCM to quantify propidium iodide (PI)- or Annexin V-positive cDC2 derived from lungs of both normal and infected mice. The findings indicated that the overall apoptosis rate (Annexin V^+^) (*P* < 0.01) and the late apoptosis rate (Annexin V^+^PI^+^) (*P* < 0.01) of cDC2 in infected mice were markedly reduced compared to the normal group (Fig. 5A). Subsequently, we isolated CD11c⁺ DCs from lungs of both normal and infected mice and examined mRNA expression levels of apoptosis-related genes including *Bcl2, Bcl2l1, Casp3, Bax, Bad*, and *Bcl2l2*. The expression of *Bcl2l1, Casp3, Bax, Bad and Bcl2l2* was not significantly different between the infected and normal mice. Nonetheless, the mRNA expression levels of autophagy-related genes *Atg5* (*P* < 0.01)*, Atg7* (*P* < 0.01), and *Becn1* (*P* < 0.01) were markedly diminished in infected mice relative to normal mice (Fig. 5B). Given that autophagy and apoptosis have intersecting signaling pathways in specific clinical contexts, our findings indicated that *Plasmodium* infection may impede cDC2 apoptosis in lungs by inhibiting autophagy.

To further elucidate the role of autophagy in cDC2 within the pulmonary environment, we subjected lung cells from mice to iRBC lysate for 3 days, subsequently conducting FCM analysis of cDC2 proportions. The findings indicated that the proportion of cDC2 in the iRBC lysate-treated group was markedly elevated compared to the untreated group (*P* < 0.001). In a subsequent experiment, iRBC lysate was administered to an additional group, followed by the introduction of the autophagy activator rapamycin (Rapa). The results showed that the proportion of cDC2 was significantly diminished relative to the Rapa-untreated group (*P* < 0.01) (Figs. 5C and S3A), In addition, compared to the Rapa-untreated group, the phagocytic activity of cDC2 increased (*P* < 0.001) (Fig. S3B), suggesting that autophagy activation could mitigate the accumulation of cDC2 caused by *Plasmodium* infection and promote its phagocytic ability.

To further explore the mechanism by which *Plasmodium* infection regulates autophagy in cDC2 of mouse lungs, we first detected the phosphorylation levels of several related transcription factors, including STAT3, p65, and AKT. The results showed that phosphorylation levels of STAT3 in cDC2 of lungs in infected mice (*P* < 0.001) was significantly higher compared to that in normal mice, while there were no significant differences in phosphorylation levels of p65 and AKT between infected and normal mice (Fig. 5D). Western blot analysis further confirmed that the expression of STAT3 and its phosphorylated form (p-STAT3) proteins in CD11c^+^ DCs of lungs in infected mice was significantly upregulated compared to that in normal mice, densitometric analysis revealed that the relative levels of p-STAT3/STAT3 was upregulated in the infected group (*P* < 0.01) (Fig. 5E). Immunofluorescence staining was performed to detect and quantify the expression of p-STAT3 in the sorted cells from both groups. The results showed that the relative density of p-STAT3 was increased in the infected group (*P* < 0.01) (Fig. 5F). Next, we treated normal mouse lung cells with uRBC lysate and iRBC lysate, respectively, and detected the phosphorylation level of STAT3 in mouse lung cDC2 by FCM. The results showed that the phosphorylation level of STAT3 in mouse lung cDC2 treated with iRBC lysate was significantly higher than that in mouse lung cDC2 treated with uRBC lysate (*P* < 0.01) (Fig. 5G).

To clarify the functional association between STAT3 and autophagy, we treated cells with iRBC lysate and the STAT3 inhibitor JSI-124, and detected autophagy-related genes by RT-qPCR. The results showed that expression levels of autophagy genes *Atg7* and *Becn1* in lung cells of the iRBC lysate treatment group were significantly inhibited compared to the control group (*P* < 0.05), while the expression of *Atg5*, *Atg7*, and *Becn1* in the iRBC lysate + JSI-124 treatment group was significantly higher than that in the iRBC lysate treatment group (*P* < 0.05) (Fig. 5H). The total apoptosis level (*P* < 0.001), early apoptosis level (*P* < 0.001), and late apoptosis level (*P* < 0.05) of mouse lung cDC2 cells in the iRBC lysate + JSI-124 treatment group were significantly higher than those in the iRBC lysate treatment group, restoring the apoptosis inhibition induced by iRBC lysate (Figs. 5I and S3C). Moreover, the proportion of IL-12-positive cDC2 in the lung of iRBC lysate + JSI-124 treated mice (*P* < 0.001) was significantly lower than that of iRBC lysate treated mice (Fig. S3D). In addition, FCM results showed that the iRBC lysate + JSI-124 treatment group effectively inhibited the increased proportion of mouse lung cDC2 induced by iRBC lysate (*P* < 0.001) (Figs. 5J and S3E). iRBC lysate + JSI-124 treatment further promoted the expression of CD86 (*P* < 0.01) in lung cDC2 of mice induced by iRBC lysates (Fig. S3F). Taken together, our results indicated that *Plasmodium* infection inhibited cDC2 apoptosis and autophagy, possibly by activating STAT3 signaling. Inhibition of STAT3 phosphorylation suppressed cDC2 proliferation.

### iRBCs promoted STAT3 phosphorylation by activating TLR3 and TLR7 on cDC2 within lungs of mice

**Fig. 6:**
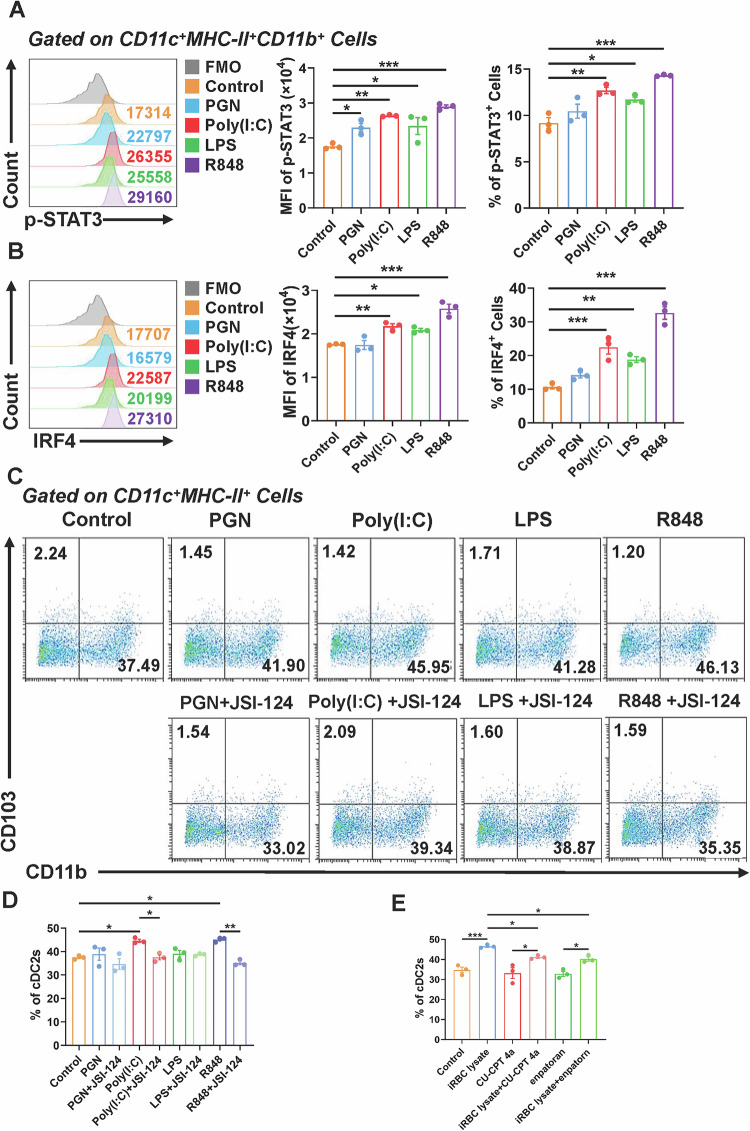
iRBCs promoted STAT3 phosphorylation by activating TLR3 and TLR7 on cDC2. The lung cells of normal mice were cultured with PGN (50 μg/mL), Poly(I: C) (50 μg/mL), LPS (50 ng/mL), R848 (100 ng/mL), and the level of p-STAT3 (**A**) and IRF4 (**B**)of pulmonary cDC2 were detected by FCM. The lung cells of normal mice were cultured with PGN, Poly(I: C), LPS, R848 and JSI-124; the percentage of cDC2 was detected by FCM (**C**, **D**). The lung cells of normal mice were cultured with iRBC lysate, CU-CPT 4a (10 μM) and Enpatoran (1 μM), and the percentage of cDC2 was detected by FCM (**E**). Date were presented as the mean ± SD and representative of three independent experiments, *, *P* < 0.05；**, *P* < 0.01；***, *P* < 0.001.

The pattern recognition receptors (PRRs) on mouse lung cDC2, such as Toll-like receptors (TLRs), are crucial in immune control and inflammatory responses. To examine whether TLRs may modulate mouse lung cDC2 through the activation of STAT3 phosphorylation, we subjected mouse lung cells to various TLR agonists in vitro. Specifically, we utilized TLR2 agonist PGN, TLR3 agonist Poly(I:C), TLR4 agonist LPS, and TLR7 agonist R848. FCM analysis demonstrated that treatments with Poly(I: C) (MFI, *P* < 0.01; proportion, *P* < 0.01) and R848 (MFI, *P* < 0.001; proportion, *P* < 0.001) significantly enhanced STAT3 phosphorylation relative to the control group, whereas treatments with PGN (MFI, *P* < 0.05; proportion, *P* > 0.05) and LPS (MFI, *P* < 0.05; proportion, *P* < 0.05) exhibited weak differences (Fig. 6A). Furthermore, we sought to evaluate whether the effect of TLRs on pulmonary cDC2 is exerted independently of STAT3 phosphorylation. We also detected the expression levels of IRF4 and p-p65, the latter being a subunit of NF-κB. The results showed that, compared with the control group, treatment with Poly(I: C) (MFI, *P* < 0.01; proportion, *P* < 0.001), LPS (MFI, *P* < 0.05; proportion, *P* < 0.01) and R848 (MFI, *P* < 0.001; proportion, *P* < 0.001) significantly enhanced the expression of IRF4, whereas no significant effect was observed on p-p65 (Figs. 6B and S3G). Additionally, we sought to ascertain if the increase in mouse lung cDC2 produced by Poly(I: C) and R848 was governed by STAT3 phosphorylation. We co-treated mouse lung cDC2 with JSI-124 and different agonists in vitro. FCM data demonstrated that the percentage of mouse lung cDC2 in the Poly(I: C) + JSI-124 (*P* < 0.05) and R848 + JSI-124 (*P* < 0.01) treatment groups was markedly diminished compared to the Poly(I: C) and R848 treatment groups, respectively (Fig. 6C, D). Nonetheless, there was no statistically significant change in the proportion of mouse lung cDC2 between the PGN + JSI-124 and LPS + JSI-124 treatment groups as compared to the PGN and LPS treatment groups, respectively. Subsequently, we added the TLR3 inhibitor CU-CPT 4a and the TLR7 inhibitor Enpatoran to the iRBCs-treated lung cells to detect the proportion of cDC2 in the lungs of mice. The results showed that the proportion of cDC2 in the lungs of mice in the iRBC lysate + CU-CPT 4a treatment group (*P* < 0.05) and the iRBC lysate + Enpatoran treatment group (*P* < 0.05) was significantly lower than that in the iRBC lysate treatment group (Figs. 6E and S3H). Our above results indicated that TLR3 and TLR7 activation contributed to the phosphorylation of STAT3, thereby promoting cDC2 proliferation. The inhibition of TLR3 or TLR7 significantly suppressed iRBC lysate-induced cDC2 accumulation within lungs.

### Inhibiting STAT3 phosphorylation reduced cDC2 accumulation in the lungs of Plasmodium-infected mice and mitigated lung damage

**Fig. 7:**
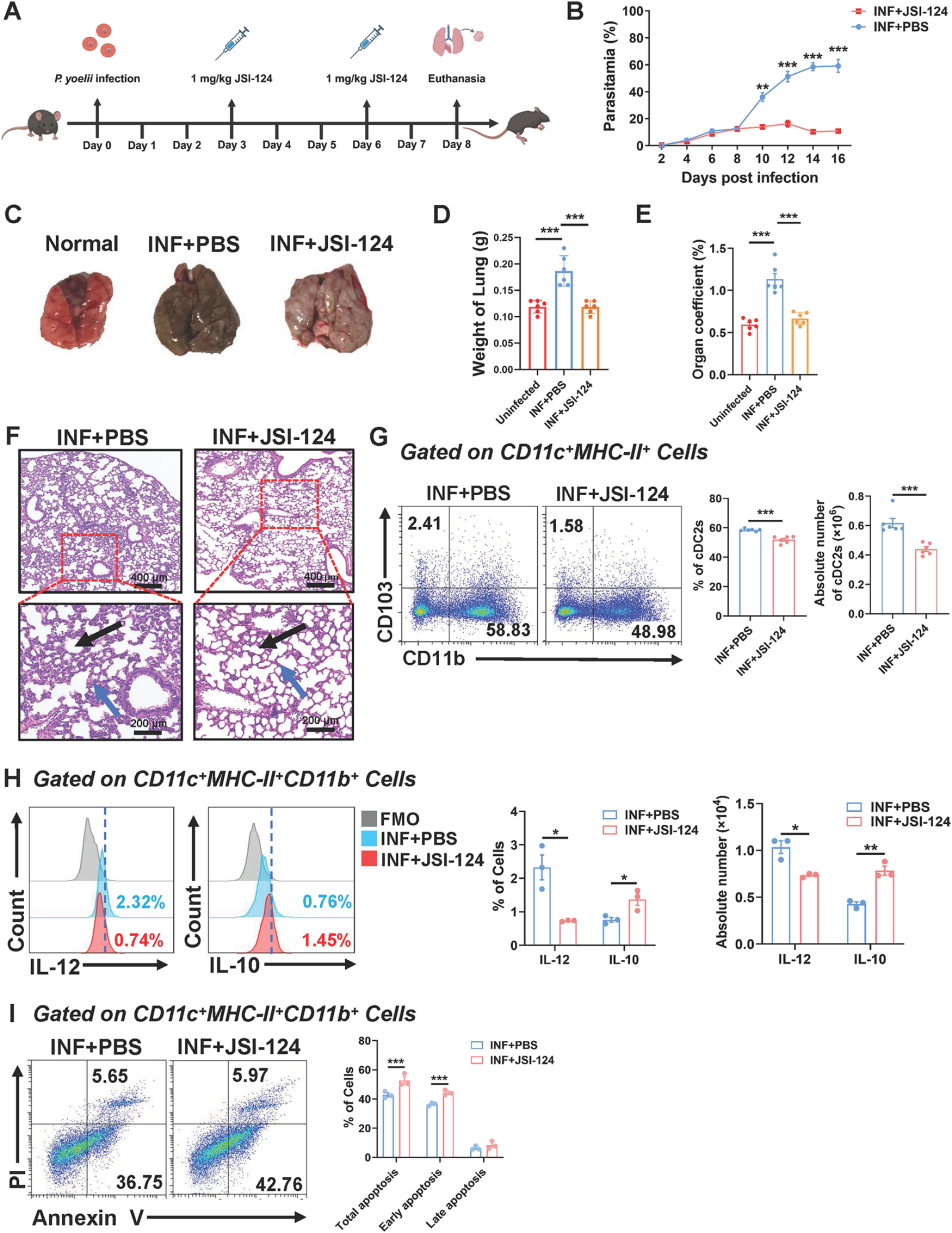
The inhibition of STAT3 phosphorylation mitigated lung injury and reduced cDC2 accumulation. Schematic representation of the JSI-124 administration model in mice (**A**). Mice were intraperitoneally injected every 3 days with 1 mg/kg of JSI-124, starting on the third day post-infection, while the control group received an equal volume of PBS. The following parameters were monitored: parasitemia (**B**). Representative images of lung tissues (**C**). The weights of the lungs (**D**) and organ coefficient (**E**) were compared among the three experimental groups. Representative images of H&E staining of lung tissue from the two experimental groups. Blue arrows indicate alveolar morphology, while black arrows point to leukocyte infiltration. Scale bars represent 400 μm and 200 μm (**F**). Mice were euthanized at 8 dpi, lung cells were collected, and the percentage and absolute number of cDC2 were determined by FCM, (*n* = 6) (**G**). FCM detection of IL-12 and IL-10 secretion levels by pulmonary cDC2 (**H**). The apoptosis of pulmonary cDC2 was detected by FCM (**I**). Date were presented as the mean ± SD and representative of three independent experiments, *, *P* < 0.05；**, *P* < 0.01；***, *P* < 0.001.

To examine the effects of STAT3 inhibition on *Plasmodium*-infected mice, we administered the STAT3 phosphorylation inhibitor JSI-124 via intraperitoneal injection on days 3 and 6 post-infection (1 mg/kg) (Fig. 7A). We assessed parasitemia rates in both INF + PBS group and INF + JSI-124 group. The findings indicated that from day 10 post-infection, the parasitemia rate in the INF + JSI-124 group was markedly lower than in the INF + PBS group and did not increase over time (Fig. 7B), implying that STAT3 inhibition successfully mitigated the advancement of parasitemia. We subsequently investigated the pathological alterations in the lungs of infected mice administered JSI-124. The lungs of INF + PBS mice exhibited significant malarial pigment accumulation and appeared darker than those of the uninfected group. In INF + JSI-124 group, these pathological alterations were mitigated, exhibiting decreased congestion and edema (Fig. 7C). The weight (*P* < 0.001) and lung coefficient (*P* < 0.001) of lungs in INF + PBS group mice were considerably reduced compared those the uninfected group, and the weight (*P* < 0.001) and lung coefficient (*P* < 0.001) of lungs in INF + JSI-124 group mice were considerably reduced compared those the INF + PBS group (Fig. 7D, E). H&E staining of lung tissues demonstrated that the destruction of alveolar structure, infiltration of inflammatory cells, and interstitial fibrosis were markedly alleviated in the INF + JSI-124 group compared to the INF + PBS group mice, suggesting that STAT3 inhibition could mitigate lung damage induced by *Plasmodium* infection (Fig. 7F).

To assess the impact of JSI-124 treatment on pulmonary cDC2 in *Plasmodium*-infected mice, we conducted FCM to examine cDC2 accumulation in the lungs of both the INF + PBS group and the INF + JSI-124 group. The findings indicated that both the proportion (*P* < 0.001) and absolute number (*P* < 0.001) of cDC2 in lungs of the INF + JSI-124 group were markedly reduced compared to the INF + PBS group (Fig. 7G). Subsequently, we evaluated the effect of JSI-124 therapy on cytokine secretion by pulmonary cDC2. FCM analysis indicated that both the proportion (*P* < 0.05) and absolute number (*P* < 0.05) of cDC2 producing IL-12 in the INF + JSI-124 group were markedly diminished compared to the INF + PBS group. Conversely, the proportion (*P* < 0.05) and absolute number (*P* < 0.01) of cDC2 producing IL-10 in the INF + JSI-124 group were markedly elevated relative to the INF + PBS group (Fig. 7H). Moreover, the total apoptosis rate (*P* < 0.001) early apoptosis (*P* < 0.001) of cDC2 in the INF + JSI-124 group were markedly elevated compared to the INF + PBS group (Fig. 7I). Taken together, our results showed that the inhibition of STAT3 phosphorylation by JSI-124 reduced the accumulation of cDC2 within lungs of infected mice and alleviated lung damage.

### Plasmodium infection facilitated the formation of pulmonary cDC2 by upregulating the expression of IRF4

**Fig. 8:**
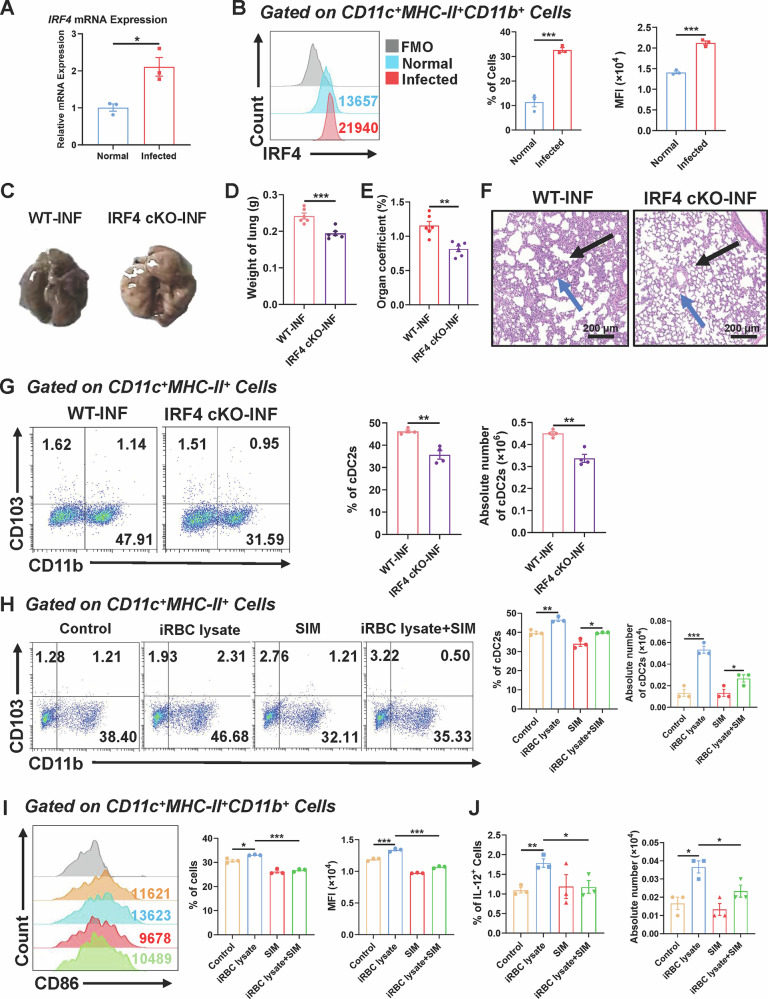
The upregulation of IRF4 contributed to pulmonary cDC2 accumulation. CD11c^+^ DCs were isolated from normal and infected mouse lung cells, and the expression of IRF4 was detected by RT-qPCR (**A**). The proportion and average fluorescence intensity of IRF4 in pulmonary cDC2 from the normal and infected mice were detected by FCM (**B**). Representative images of lung tissues (**C**). The weights of the lungs (**D**) and organ coefficient (**E**) were compared between the two experimental groups. Representative images of H&E staining of lung tissues. Blue arrows indicate alveolar morphology, while black arrows point to leukocyte infiltration. Scale bars represent 200 μm (**F**). At 8 days post-infection, mice were euthanized, lung cells were collected, and the percentage and absolute number of cDC2 were determined by FCM, (*n* = 6) (**G**). Single cell suspension was isolated from the lungs of normal mice and treated with iRBC lysate and SIM (**H**). Measure the proportion and absolute number of cDC2, activated molecule CD86 (**I**), and cytokine IL-12 (**J**) with or without the addition of IRF4 inhibitor SIM(1 μg/mL). Date were presented as the mean ± SD and representative of three independent experiments, *, *P* < 0.05；**, *P* < 0.01；***, *P* < 0.001.

IRF4 is a crucial transcription factor that governs the differentiation and development of cDC2 in the lung of mice. To examine the function of IRF4 in cDC2 within the pulmonary tissue of mice during *Plasmodium* infection, we isolated CD11c^+^ DCs from lungs of both normal and infected mice and assessed IRF4 expression with RT-qPCR. The findings indicated that the expression level of *Irf4* in CD11c^+^ DCs from lungs of infected mice was markedly elevated compared to that in control mice (*P* < 0.05) (Fig. 8A). Subsequently, we assessed the expression level of IRF4 within cDC2 in lungs of both normal and infected mice utilizing FCM. Our results demonstrated that the percentage (*P* < 0.001) and MFI (*P* < 0.001) of IRF4 in cDC2 from lungs of infected mice were markedly elevated compared to normal mice (Fig. 8B). To elucidate the effects of IRF4 deficiency on *Plasmodium*-infected mice, we generated myeloid-specific conditional knockout (cKO) mice lacking the *Irf4* gene. Both wildtype (WT) and IRF4 cKO mice were inoculated with equivalent quantities of *Plasmodium*-infected erythrocytes. On day 14 following infection, mice were euthanized to harvest lung tissues. In comparison to WT-INF mice, lungs of IRF4 cKO-INF mice had less pigment deposition, congestion, and edema (Fig. 8C). Moreover, the wet weight (*P* < 0.001) and lung coefficient (*P* < 0.01) of lungs from IRF4 cKO-INF mice were markedly reduced compared to those from WT-INF mice (Fig. 8D, E). Subsequently, we conducted H&E staining on lung tissues from WT-INF and IRF4 cKO-INF mice. The findings indicated significant alveolar fusion and inflammatory cell accumulation in the lung tissues of WT-INF mice, accompanied by morphological disarray and thickening of the alveolar cavity (Fig. 8F). Conversely, the lung architecture of IRF4 cKO-INF mice exhibited greater regularity, and the aggregation of inflammatory cells was less, suggesting that IRF4 cKO mitigated lung damage induced by *Plasmodium* infection in mice.

To assess the effects of IRF4 loss on pulmonary cDC2, we utilized FCM to examine the accumulation of cDC2 in lungs of WT and IRF4 cKO mice infected with *Plasmodium*. Results revealed a notable reduction in both the proportion (*P* < 0.01) and absolute number (*P* < 0.01) of cDC2 in lungs of IRF4 cKO-INF mice relative to WT-INF mice (Fig. 8G). Furthermore, following the treatment of mice lung cells with the IRF4 inhibitor simvastatin (SIM) alongside iRBC lysate, we evaluated the proportion and absolute number of pulmonary cDC2 utilizing FCM. The data indicated that both the proportion (*P* < 0.01) and absolute number (*P* < 0.01) of cDC2 in the lungs of the iRBC lysate +SIM treatment group were considerably reduced compared to the iRBC lysate treatment group (Fig. 8H). Moreover, the expression proportion (*P* < 0.001) and MFI (*P* < 0.001) of the activation marker CD86 on cDC2 in lungs of mice subjected to the iRBC lysate +SIM therapy were significantly diminished in comparison to the group treated with iRBC lysate only (Fig. 8I). Furthermore, the proportion (*P* < 0.05) and absolute number (*P* < 0.05) of cDC2 producing IL-12 in lungs of mice subjected to the iRBC lysate +SIM therapy were markedly reduced compared to the group treated with iRBC lysate only (Fig. 8J). These findings indicated that IRF4 intensified pulmonary immunopathological damage in *Plasmodium*-infected mice by facilitating the growth, activation, and release of the pro-inflammatory cytokine IL-12 from pulmonary cDC2.

### Plasmodium infection modulated the autophagy of pulmonary cDC2 via the STAT3-IRF4 signaling pathway

**Fig. 9:**
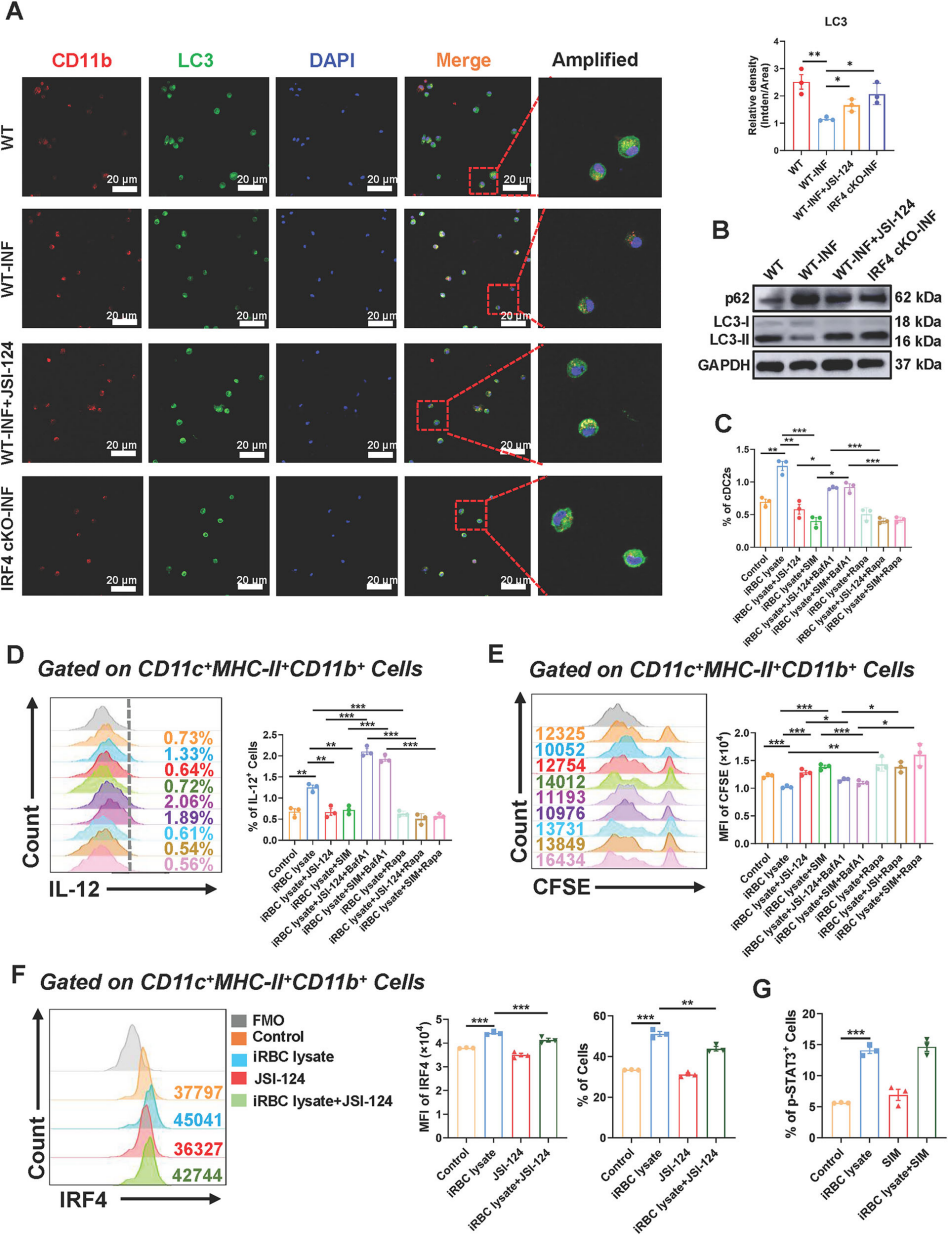
*Plasmodium* infection modulated pulmonary cDC2 autophagy via STAT3-IRF4 signaling. CD11c^+^ DCs from the lungs were sorted for immunofluorescence detection (**A**) and western blot (**B**). Single-cell suspensions were isolated from the lungs of normal mice and treated with iRBC lysis. After treatment with JSI-124 (500 nM), SIM (1 μg/mL), BafA1 (100 nM) and Rapa(5 nM), the proportion (**C**) of cDC2, cytokine IL-12 production (**D**), and phagocytic ability (**E**) were measured. The lung cells of normal mice were cultured with iRBC lysates and JSI-124, the expression of IRF4 was detected by FCM (**F**). The lung cells of normal mice were cultured with iRBC lysates and SIM, the expression of p-STAT3 was detected by FCM (**G**). Date were presented as the mean ± SD and representative of three independent experiments, *, *P* < 0.05；**, *P* < 0.01；***, *P* < 0.001.

To clarify the functions of STAT3 and IRF4 in modulating autophagy of cDC2 in the lung of mice, we isolated CD11c^+^ DCs from lungs of WT mice, WT-INF mice, WT-INF mice treated with JSI-124, and IRF4 cKO-INF mice. Thereafter, sorted cells were cultured in 24-well plates. Immunofluorescence detection using primary antibodies targeting CD11b and LC3. LC3 is a crucial autophagy-related protein that modulates autophagy activity by translational modification. Hence, LC3 expression is typically regarded as an indicator of autophagy occurrence. Immunofluorescence data showed that the relative density of LC3 was decreased in the WT-INF group compared to the WT group (*P* < 0.01). Administration of JSI-124 in WT-infected mice (*P* < 0.05) and IRF4 cKO-infected mice (*P* < 0.05) enhanced LC3 expression in lung cDC2 compared to the WT-INF group (Fig. 9A), indicating that STAT3 and IRF4 modulated autophagy in lung cDC2. Subsequently, we evaluated sorted cells utilizing western blot analysis. The findings indicated that p62 expression was elevated in WT-INF mice relative to WT mice, while p62 expression was decreased in both WT-INF + JSI-124 mice and IRF4 cKO-INF mice compared to WT-INF mice. p62 is a crucial protein in autophagy, binding to ubiquitinated proteins to facilitate their entry into autophagosomes, which then fuse with lysosomes to create autophagolysosomes for degradation. Inhibition of autophagic flow results in elevated p62 levels, while activation of autophagic flux leads to a reduction in p62 levels. In addition, LC3-II expression was diminished in WT-INF mice relative to WT mice, while LC3-II expression was elevated in WT-INF + JSI-124 mice and IRF4 cKO-INF mice compared to WT-INF mice (Fig. 9B). The elevation of LC3-II signifies the buildup of autophagosomes within the cytoplasm of cells. The aforementioned results indicated that autophagy in cDC2 within lungs of WT-INF mice was suppressed, while autophagy was augmented in WT-INF + JSI-124 mice and IRF4 cKO-INF mice compared to WT-INF mice, further demonstrating the regulatory role of STAT3 and IRF4 on cDC2 autophagy in lungs of mice.

Subsequently, we sought to examine whether STAT3 and IRF4 could modulate the accumulation of cDC2 in pulmonary tissues of mice via autophagy. We administered iRBC lysate to normal mouse lung cells in vitro and assessed the fraction of cDC2 in lungs of mice following treatment with JSI-124 and SIM, in conjunction with Rapa and the autophagy inhibitor Bafilomycin A1 (BafA1). The findings indicated that the percentage of cDC2 in lungs of mice subjected to the iRBC lysate + JSI-124 + BafA1 therapy was markedly greater than that in the iRBC lysate + JSI-124 treatment group (*P* < 0.01). There was no significant difference between the iRBC lysate + JSI-124 + rapamycin treatment group and the iRBC lysate + JSI-124 treatment group. The percentage of cDC2 in lungs of mice subjected to the iRBC lysate + SIM + BafA1 treatment was significantly elevated compared to the iRBC lysate + SIM treatment group (*P* < 0.05), and there was no significant difference between the iRBC lysate + SIM + rapamycin treatment group and the iRBC lysate + SIM treatment group, suggesting that BafA1 could counteract the suppressive influence of STAT3 or IRF4 on cDC2 accumulation (Figs. 9C and S4A). Additionally, we aimed to investigate whether STAT3 and IRF4 may modulate the activity of cDC2 in the pulmonary environment of mice via autophagy. FCM revealed that the capacity of cDC2 in lungs of mice to secrete IL-12 was markedly elevated in the iRBC lysate + JSI-124 + BafA1 treatment group compared to the iRBC lysate + JSI-124 treatment group (*P* < 0.001) (Fig. 9D). Conversely, the phagocytic capability of cDC2 in lungs of mice in the iRBC lysate + JSI-124 + BafA1 treatment group was significantly diminished relative to the iRBC lysate + JSI-124 treatment group (*P* < 0.05) (Fig. 9E). The secretion of IL-12 by cDC2 in lungs of mice was markedly elevated in the iRBC lysate + SIM + BafA1 treatment group compared to the iRBC lysate + SIM group (*P* < 0.001). Conversely, the phagocytic capacity of cDC2 in the iRBC lysate + SIM + BafA1 group was significantly diminished relative to the iRBC lysate + SIM group (*P* < 0.001). These results suggested that BafA1 might mitigate the inhibitory effects of STAT3 or IRF4 on the functionality of cDC2 in mouse lungs.

To investigate the regulatory interaction between STAT3 and IRF4, we subjected normal mice lung cells to iRBC lysate in vitro. Following a 3-day treatment with JSI-124 and SIM, we quantified the expression of IRF4 and the phosphorylation of STAT3 in pulmonary cDC2 via FCM. The findings indicated that the proportion (*P* < 0.01) and MFI (*P* < 0.001) of IRF4 in pulmonary cDC2 were markedly reduced in the JSI-124 combined with iRBC lysate treatment group relative to the iRBC lysate treatment group (Fig. 9F). Nonetheless, there was no statistically significant difference in the proportion of STAT3 phosphorylation in pulmonary cDC2 between the SIM combined with iRBC lysate treatment group and the iRBC lysate treatment group, suggesting that STAT3 functioned upstream of IRF4 and modulated IRF4 expression (Figs. 9G and S4B). In conclusion, *Plasmodium* infection modulated the autophagy of cDC2 within mouse lungs via the STAT3-IRF4 signaling pathway.

## Discussion

**Fig. 10:**
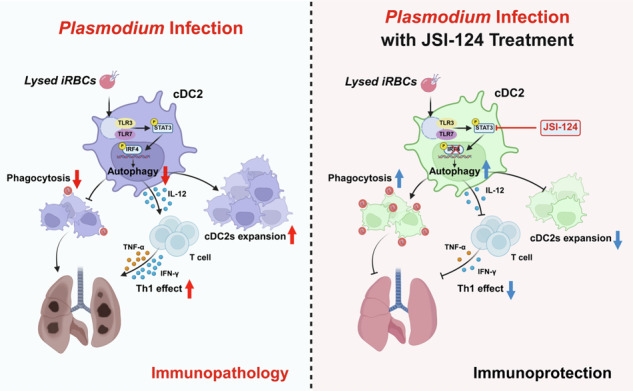
Schematic of *Plasmodium* infection-induced pulmonary cDC2 accumulation. *Plasmodium* infection inhibited lung cDC2 autophagy through the TLR3/7-STAT3-IRF4 signaling pathway, resulting in pathological damage and compromised parasite clearance within mouse lungs. Elevated IL-12 secreted by cDC2 promoted Th1 differentiation, which induced detrimental pulmonary inflammation. Targeting STAT3 or IRF4 restored cDC2 autophagy, crippled their abnormal expansion, and mitigated Th1 cell-mediated inflammation, thereby attenuating lung damage.

Malaria is a dangerous parasite disease that threatens human health and safety. Humans have made incredible progress in the fight against malaria: Artemisinin combination treatment (ACTs) has saved tens of millions of lives. ACTs were a watershed moment in worldwide malaria prevention and control, but in recent years, artemisinin resistance has arisen in Southeast Asia and Africa, posing a significant concealed threat [2]. In this study, we found that *Plasmodium* infection inhibits cDC2 autophagy in the mouse lung via the TLR3/7-STAT3-IRF4 pathway, favoring *Plasmodium* infection and excessive immunological inflammation. In contrast, suppressing STAT3 phosphorylation or IRF4 expression triggers cDC2 autophagy in the mouse lung, reducing inflammatory cytokine production, relieving lung inflammation, and slowing *Plasmodium* infection progression (Fig. 10). Our findings highlight the critical limiting roles of transcription factors STAT3 and IRF4, as well as autophagy, in the pathogenesis of *Plasmodium* infection and suggest possible antimalarial treatments.

Malaria patients who do not receive timely treatment may suffer catastrophic repercussions, including pulmonary edema [32]. In our work, we found that *P. y*17XNL infection induced lung harm in mice. Indeed, the lung serves as both a respiratory and immunological organ. The body forms a large lymphatic network along the alveolar wall to keep the lung immunological microenvironment stable [33]. Among them, cDC2 is a critical antigen-presenting cell, and its immunological regulatory function in asthma and infectious illnesses has been widely elucidated [7]. In this investigation, we detected cDC2 accumulation in the lungs of mice, which is consistent with the finding that parasite infection can induce cDC2 growth in response to a variety of pathogenic microorganisms [34]. After detecting changes in homeostasis caused by inflammatory signals from pathogens or tissues, cDC2 switches from a quiescent to an active state [35], at which point immature DCs have a high phagocytic capacity [36], and iRBCs express PfEMP on their surface, which is required for immune evasion and cell adhesion. DCs can directly engage with iRBCs via surface receptors, promoting phagocytosis [37]. VCAM-1, PECAM-1, ICAM-1, CD209a, and CD36 are key adhesion molecules on DCs. CD36 is found on other immune cells, such as DCs and enhances the capture of iRBCs, which improves immune cell phagocytosis against *Plasmodium* [38]. Furthermore, it can inhibit phagocytosis by expressing ROS and NO [39, 40]. This phagocytic capacity will drop as it matures, transitioning from “catcher” to “antigen presenter,” activating T cells [41]. For example, in anti-tumor immunity, cDC2 secretes inflammatory cytokine IL-12, causing CD4^+^ T cells to differentiate into effector Th1 cells and mount an anti-tumor immune response [42]. In our study, immature cDC2 in mouse lungs matured gradually after identifying the expression of CD80 and CD86, molecules activated by *Plasmodium* signals. During this process, its ability to phagocytose and eliminate *Plasmodium* steadily reduced, promoting the proliferation, maturation, and differentiation of T cells into Th1 to perform the type 1 immune response.

The association between apoptosis and DCs has been extensively researched [31], but the relevance of apoptosis in lung cDC2 in *Plasmodium* infection remains to be investigated. Interestingly, we discovered that lung cDC2 apoptosis was reduced following *Plasmodium* infection. After infection, autophagy-related genes *Atg5*, *Atg7*, and *Becn1* were considerably downregulated, although apoptosis-related genes *Bax*, *Bad*, and *Casp3* did not alter significantly. This phenomenon is similar to the regulatory effect of autophagy on apoptosis [43]. One possible explanation for the inhibition of cDC2 apoptosis in the lung following infection, with no distinct changes in apoptotic genes and downregulation of autophagy-related genes, is that apoptosis and autophagy signaling pathways cross-regulate. *Plasmodium* infection inhibits autophagy, which reduces cDC2 apoptosis in the lung and promotes its accumulation. For example, excessive autophagy activation has been shown to induce apoptosis via Beclin-1 regulation of essential components such as Caspases [44]. Furthermore, rapa has been shown to increase autophagy by blocking the activity of mTOR. Our results demonstrated that rapa might suppress the phenomenon of iRBC lysate-induced lung cDC2 accumulation. This is consistent with previous findings that rapa promotes tumor cell proliferation and autophagy in the tumor environment [45]. As a result, our findings imply that *Plasmodium* infection may regulate cell death via autophagy, leading to the buildup of cDC2 in the lung.

The JAK2-STAT3 signaling pathway is involved in the control of DCs immunological responses [46, 47]. Our research discovered that cDC2 in the infected lung might enhance STAT3 phosphorylation, which was accompanied by an aggravation of pathological conditions in the lungs of mice. Similarly, earlier research has demonstrated that optic nerve phosphatase can inhibit the downstream transcription of IL-10 by disrupting the JAK2-STAT3 of DCs. STAT3 phosphorylation inhibition promotes the course of experimental autoimmune encephalomyelitis in a mouse model [48]. Our study also discovered that inhibiting STAT3 phosphorylation will decrease the downregulation of apoptosis in lung cDC2 treated with iRBC lysate, hence inhibiting the accumulation of cDC2. In our recent work, phosphorylation suppression of STAT3 inhibited the accumulation and anti-apoptotic properties of MDSCs generated by *Plasmodium*-infected serum, and MDSCs activity was considerably reduced following JSI-124 therapy [49]. A study confirmed that knocking down STAT3 increased metformin-induced apoptosis and autophagy [50]. In this study, we discovered that STAT3 phosphorylation inhibition increased autophagy in lung cells. Notably, as a canonical transcription factor, STAT3 has been demonstrated to bind to the promoter region of the *Atg7* gene and upregulate expression in melanoma cells [51]. However, in the *Plasmodium* infection model established in this study, we found that JSI-124 mediated inhibition of STAT3 phosphorylation significantly restored the expression levels of both *Atg7* and *Becn1* genes. To date, no studies have reported a direct inhibitory effect of STAT3 on gene expression. Based on these findings, we hypothesize that STAT3 does not directly suppress the expression of genes related to autophagy. Instead, when STAT3 accumulates at the promoter regions of *Atg7* and *Becn1*, it may transcriptionally activate other downstream genes that can inhibit the expression of these two genes. Although the screening and validation of such genes are beyond the scope of the present study, further in-depth investigations are warranted in subsequent research. Furthermore, during the immunological response to invading pathogens, mice DCs express a variety of TLRs [52]. We discovered that iRBC can enhance STAT3 phosphorylation and cDC2 accumulation in the lung via upregulating TLR3 and TLR7 expression. As a result, our findings demonstrated that lung cDC2 might phosphorylate STAT3 via TLR3 and TLR7, block autophagy and apoptosis, and enhance cDC2 accumulation in the lung during *Plasmodium* infection. Furthermore, aberrant activation of the STAT3 signaling pathway is strongly related with the progression and treatment resistance of many malignant tumors, and high phosphorylation levels may be an independent risk factor for poor prognosis [53, 54]. For example, studies have shown that blocking STAT3 phosphorylation at tyr705 can effectively prevent the formation of melanoma lung metastases [55, 56]. Similarly, we discovered that mice treated with JSI-124, a STAT3 phosphorylation inhibitor, had enhanced lung function and had a significantly lower infection rate. Furthermore, mice injected with JSI-124 efficiently decreased the ability of lung cDC2 to release IL-12. As a result, we hypothesize that JSI-124 alters STAT3 phosphorylation, inhibiting the ability of lung cDC2 to produce the pro-inflammatory cytokine IL-12 and therefore mitigating mouse lung damage. These data clearly demonstrated the importance of inhibiting STAT3 phosphorylation in limiting *Plasmodium* infection and protecting the lung. However, the effect of JSI-124 on *Plasmodium* clearance in infected mice needs to be investigated further.

The transcription factor IRF4 is required for controlling moDCs lineage development, particularly for the creation and functional maintenance of cDC2 subpopulations [27, 28]. Previous research has demonstrated that IRF4 expression is needed for the immunomodulatory ability of lung cDC2 in the context of chronic bacterial infections and cancer [57]. In our work, we discovered that cDC2 in the lungs after infection expressed higher amounts of IRF4, and the accumulation of cDC2 in the lungs of infected mice with IRF4 cKO was much lower than that of WT mice. These findings indicate that IRF4 plays a key role in the buildup of cDC2 in mouse lungs. Furthermore, our work discovered that IRF4 loss could considerably ameliorate lung lesions and suppress IL-12 release, which could explain why IRF4 cKO mice have improved lung conditions. Furthermore, we discovered that IRF4 deletion promotes cDC2 autophagy in the mouse lung. As a result, we identified IRF4 as an essential regulator of autophagy in cDC2. Similarly, we know that STAT3 regulates autophagy, but the regulatory connection between STAT3 and IRF4 remains unknown. The current study found a connection between STAT3 and IRF4. For example, in macrophages, STAT3 regulates IRF4 transcription via the H3K27 demethylase JMJD3, resulting in M2 macrophage polarization [58]. In our investigation, we also discovered that lung cDC2 regulated IRF4 expression via STAT3 in the presence of infected erythrocytes. Furthermore, STAT3 and IRF4 can interfere with lung cDC2 activity by inhibiting autophagy. As a result, we investigated the relationship between STAT3 and IRF4 in lung cDC2 and discovered that both STAT3 and IRF4 play critical roles in autophagy regulation. However, while we have found that inhibiting STAT3 and IRF4 improves the pathological state of mice, the effect of autophagy on the infectious condition of mice needs to be explored.

In conclusion, our findings highlighted the protective role of autophagy in the pathogenesis of *Plasmodium* infection and elucidated how *Plasmodium* infection impacts autophagy and then cDC2 function via the TLR3/7-STAT3-IRF4 signaling axis. Specifically, numerous inhibitors were used in our investigation, including JSI-124, a STAT3 phosphorylation inhibitor, and SIM, an IRF4 inhibitor. JSI-124 is a specific inhibitor of the JAK/STAT3 pathway that has demonstrated significant efficacy in tumor studies [59, 60]. SIM is primarily utilized in the therapeutic treatment of primary hypercholesterolemia, but it is also increasingly used as an IRF4 inhibitor [61]. Both can reduce lung harm induced by *Plasmodium* infection by reducing the generation of the inflammatory cytokine IL-12, which is released by lung cDC2. These inhibitors may influence the development of potential treatment strategies to target lung cDC2 and reduce the detrimental effects of *Plasmodium* infection on the body, thereby contributing to the functional cure of *Plasmodium*-infected bodies.

## Conclusion

In conclusion, our research has improved the comprehension of the immune response in the pulmonary systems of mice during *Plasmodium* infection. Our research indicates that the STAT3-IRF4 signaling pathway facilitates the accumulation of cDC2 in the lungs by inhibiting their autophagy during infection. Inhibiting the phagocytic capacity of cDC2 during infection enhances Th1 differentiation, worsening lung injury. Our research findings offer immunological insights into the pulmonary symptoms associated with infection and may guide future investigations focused on developing vaccines for this neglected tropical disease.

## Supplementary information

Supporting information

Western blot raw

## Data Availability

The data of this investigation may be acquired from the corresponding author upon an appropriate demand.
