# Supplementary material for: Plasmodium yoelii infection induces lung injury by modulating type 2 conventional dendritic cells autophagy via the STAT3-IRF4 signaling
Source: Cell Death Dis. 2026 Apr 10;17(1):461. doi: 10.1038/s41419-026-08675-4 (PMC13181017; doi:10.1038/s41419-026-08675-4)
Supplement: Supplementary file 1 — Supporting information [file 41419_2026_8675_MOESM1_ESM.docx]

**Supporting information**


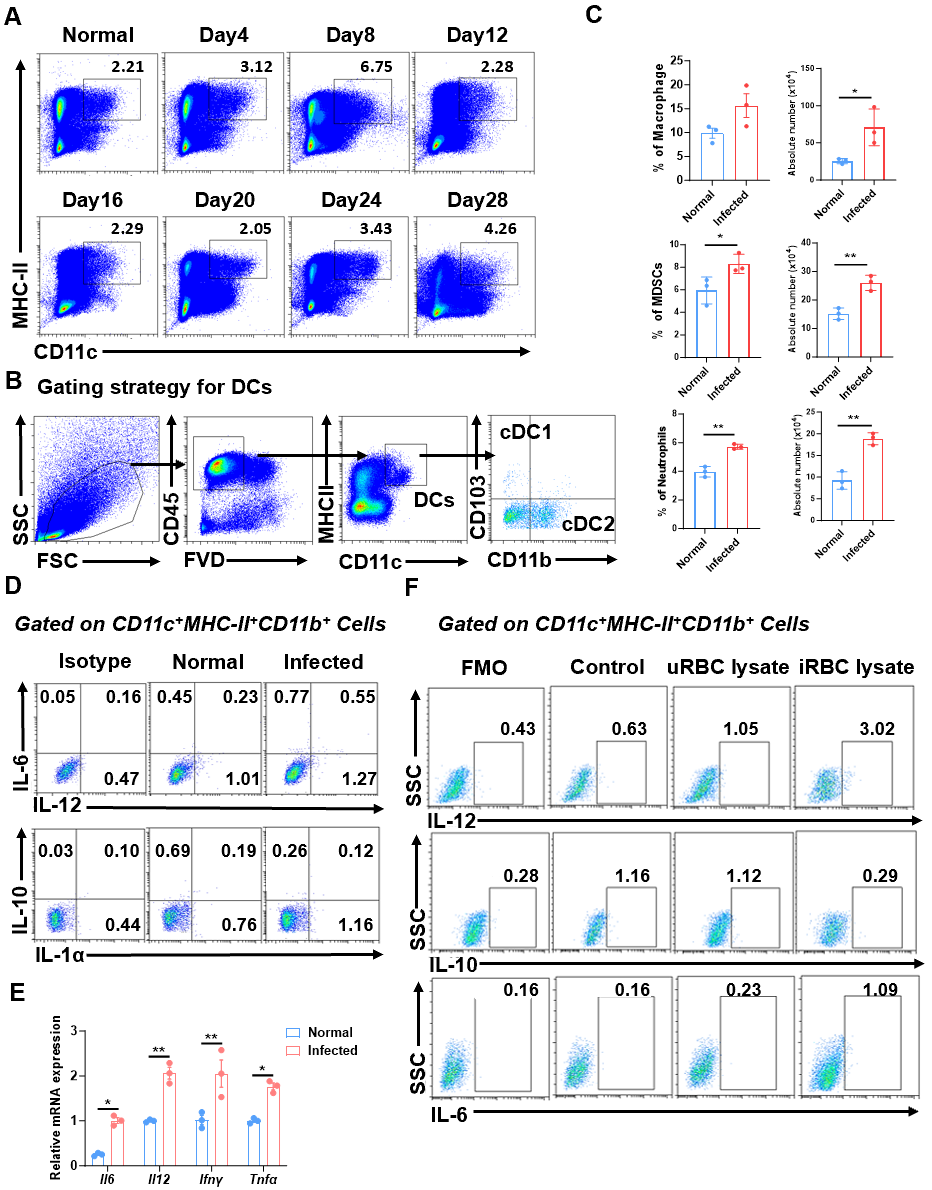


**Fig. S1. *Plasmodium* infection induced the increase of cDC2 in mouse lungs.**

(A) Flow cytometry representative plots for dynamic monitoring of DCs proportion in mouse lungs before and after infection. (B) Gating strategy used to define cDC2 in the mouse lung. Cells were isolated from grinded mouse lung. Live cells were gated on the basis of FSC and SSC parameters and doublets were excluded using FSC-A and FSC-H. Then Total lymphocytes were identified by CD45 and FVD staining. cDC2 were identified by subsequent gating as CD11c^+^MHCII^+^CD11b^+^CD103^-^cells, while cDC1 were defined as CD11c^+^MHCII^+^CD11b^-^CD103^+^ cells. (C) Statistical charts of the proportion and absolute number of macrophages, MDSCs and neutrophils in the lungs of mice in the normal group and infected group. (D) Flow cytometry representative images of cytokine secretion by pulmonary cDC2 of normal and infected mice. (E) Statistical graph of cytokine genes in sorted lung CD11c^+^cells from normal and infected mice detected by RT-qPCR. (F) Flow cytometry representative images of cytokine secretion by pulmonary cDC2 after treatment with uRBC lysate and iRBC lysates.


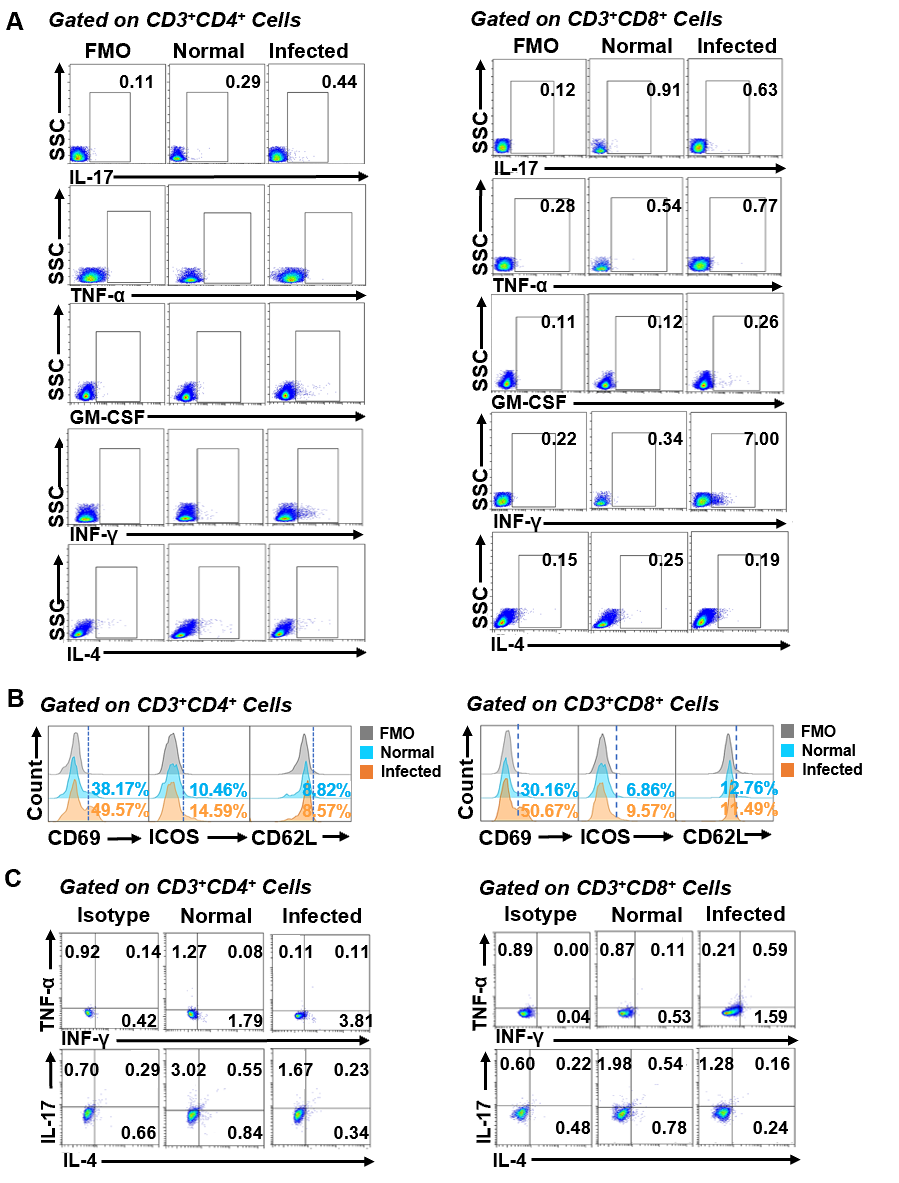


**Fig. S2. *Plasmodium* infection promoted the activation of CD4^+^ and CD8^+^ T cells.** (A) Flow cytometry representative images of cytokine secretion by CD4^+^T and CD8^+^T cells in lungs of normal and infected mice. (B) Flow cytometry representative images of CD4^+^T and CD8^+^T cell activation molecule expression in lungs of normal and infected mice. (C) Representative flow cytometry image of cytokines secreted by CD4^+^T and CD8^+^T cells co-cultured with sorted lung CD11c^+^ cells.


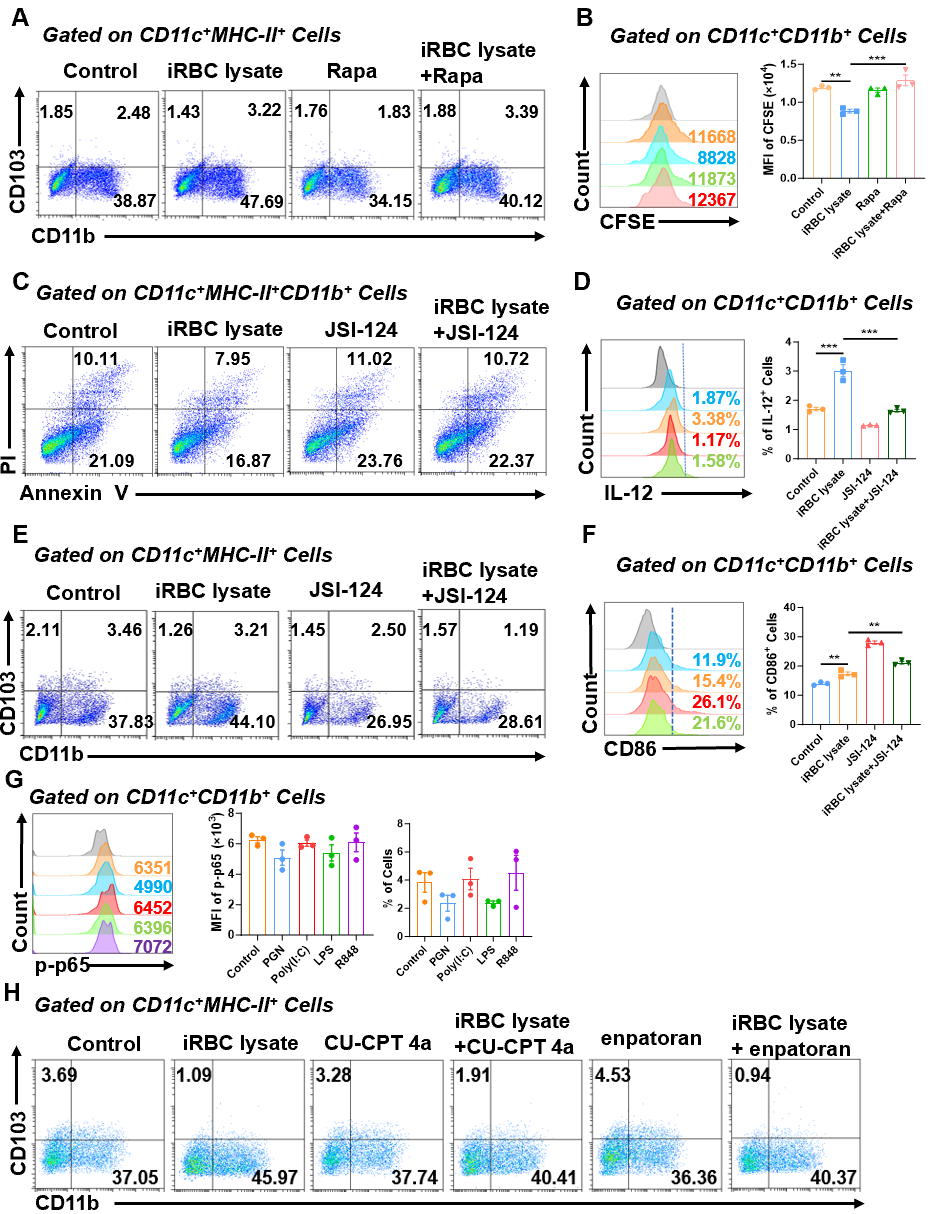


**Fig. S3. JSI-124 inhibited the activation and IL-12 secretion of cDC2.** (A) Representative flow cytometry image of detecting the proportion of cDC2 in lung cells treated with rapamycin and iRBC lysate in *vitro*. (B) Representative images and statistical graphs of detecting the phagocytic ability of cDC2 to iRBCs using rapamycin and iRBCs lysate treated lung cells in *vitro*. (C) Representative images of cDC2 apoptosis detection in lung cells treated with JSI-124 and iRBC lysate in *vitro*. (D) Representative images and statistical graphs of detecting the level of IL-12 secreted by cDC2 using JSI-124 and iRBC lysate to treat lung cells in *vitro*. (E) Representative images of detecting the proportion of cDC2 in lung cells treated with JSI-124 and iRBC lysate in *vitro*. (F) Representative images and statistical graphs of cDC2 activation marker CD86 detection in lung cells treated with JSI-124 and iRBC lysate in *vitro*. (G) The MFI and proportion of p65 expressed by cDC2 after in *vitro* treatment with different TLR agonists. (H) Representative flow cytometry images of cDC2 proportion detection in lung cells treated with CU-CPT 4a, enpatoran and iRBC lysate in *vitro*.

**
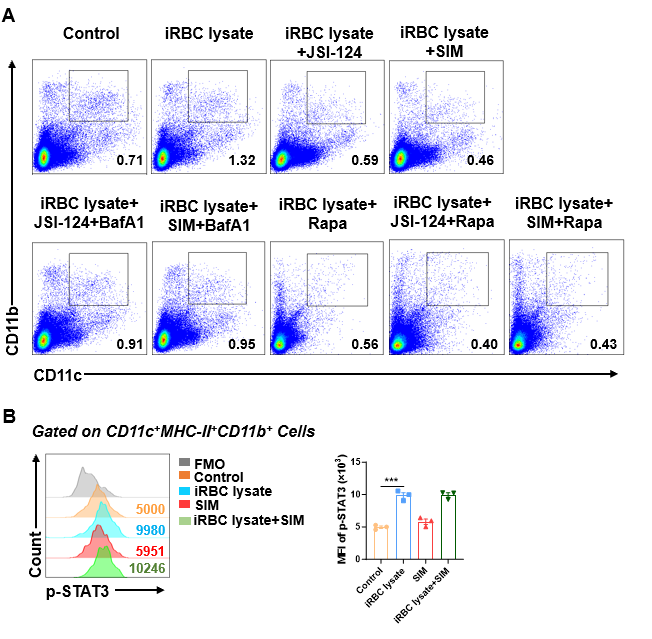
**

**Fig. S4. BafA1 antagonized JSI-124- and simvastatin-induced pulmonary cDC2 reduction.** (A) Flow cytometry representative images of cDC2 detection in lung cells treated with JSI-124, simvastatin, BafA1, rapamycin and iRBC lysate in *vitro*. (B) Representative flow cytometry images and statistical graphs of STAT3 phosphorylation detection in lung cells treated with simvastatin and iRBC lysate in *vitro*.

**Table S1. Sequences of primers used in this manuscript.**

| ***Gene*** | **Forward Primer (5'-3')** | **Reversed Primer (5'-3')** |
| --- | --- | --- |
| ***Irf4*** | AGACCAGACTTGCAAGCTCT | CACCAAAGCACAGAGTCACC |
| ***Vcam1*** | AGTTGGGGATTCGGTTGTTCT | CCCCTCATTCCTTACCACCC |
| ***Pecam1*** | ACGCTGGTGCTCTATGCAAG | TCAGTTGCTGCCCATTCATCA |
| ***Icam1*** | GTGATGCTCAGGTATCCATCCA | CACAGTTCTCAAAGCACAGCG |
| ***Cd209a*** | CTGGCGTAGATCGACTGTGC | AGACTCCTTGCTCATGTCAATG |
| ***Cd36*** | ATGGGCTGTGATCGGAACTG | GTCTTCCCAATAAGCATGTCTCC |
| ***Bcl2*** | GTCGCTACCGTCGTGACTTC | CAGACATGCACCTACCCAGC |
| ***Bcl2l1*** | CGTGGAAAGCGTAGACAAGG | GCTGCATTGTTCCCGTAGAG |
| ***Casp3*** | TCCCGTAGAGATCCACAAAAGT | TTGCTCCCATGTATGGTCTTTAC |
| ***Casp8*** | AACTGCGTTTCCTACCGAGA | CCTTGTTCCTCCTGTCGTCT |
| ***Fas*** | GAGGCCCATTTTGCTGTCAA | TTCCCTTCTGTGCATGAGGT |
| ***Myc*** | ATGCCCCTCAACGTGAACTTC | CGCAACATAGGATGGAGAGCA |
| ***Tak1*** | GACTCTGCGGTAGCCTCAC | AGGATGAACTGCTGTTTAGAGGA |
| ***Akt3*** | TGGGTTCAGAAGAGGGGAGAA | AGGGGATAAGGTAAGTCCACATC |
| ***Maf*** | GGAGACCGACCGCATCATC | TCATCCAGTAGTAGTCTTCCAGG |
| ***Bax*** | TGAAGACAGGGGCCTTTTTG | AATTCGCCGGAGACACTCG |
| ***Bad*** | AAGTCCGATCCCGGAATCC | GCTCACTCGGCTCAAACTCT |
| ***Mcl1*** | AAAGGCGGCTGCATAAGTC | TGGCGGTATAGGTCGTCCTC |
| ***Bcl2l2*** | GCGGAGTTCACAGCTCTATAC | AAAAGGCCCCTACAGTTACCA |
| ***Atg5*** | TGTGCTTCGAGATGTGTGGTT | GTCAAATAGCTGACTCTTGGCAA |
| ***Atg7*** | GTTCGCCCCCTTTAATAGTGC | TGAACTCCAACGTCAAGCGG |
| ***Becn1*** | TCCAGAGAGCACCCAATTTACC | CTGTCACCATTGTCCTCAAAGT |
| ***Il-12a*** | CCCTTGCCCTCCTAAACCAC | AAGGAACCCTTAGAGTGCTTACT |
| ***Il-6*** | CCAAGAGGTGAGTGCTTCCC | CTGTTGTTCAGACTCTCTCCCT |
| ***Ifng*** | ATGAACGCTACACACTGCATC | CCATCCTTTTGCCAGTTCCTC |
| ***Tnfa*** | GACGTGGAACTGGCAGAAGAG | TTGGTGGTTTGTGAGTGTGAG |
| ***Actb*** | ACTGCTCTGGCTCCTAGCAC | ACATCTGCTGGAAGGTGGAC |
| ***18s rRNA*** | GGGGATTGGTTTTGACGTTTTTGCG | AAGCATTAAATAAAGCGAATACATCCTTAT |
