# Supplementary figures and images for: Plasmodium yoelii infection induces lung injury by modulating type 2 conventional dendritic cells autophagy via the STAT3-IRF4 signaling
Source: Cell Death Dis. 2026 Apr 10;17(1):461. doi: 10.1038/s41419-026-08675-4 (PMC13181017; doi:10.1038/s41419-026-08675-4)

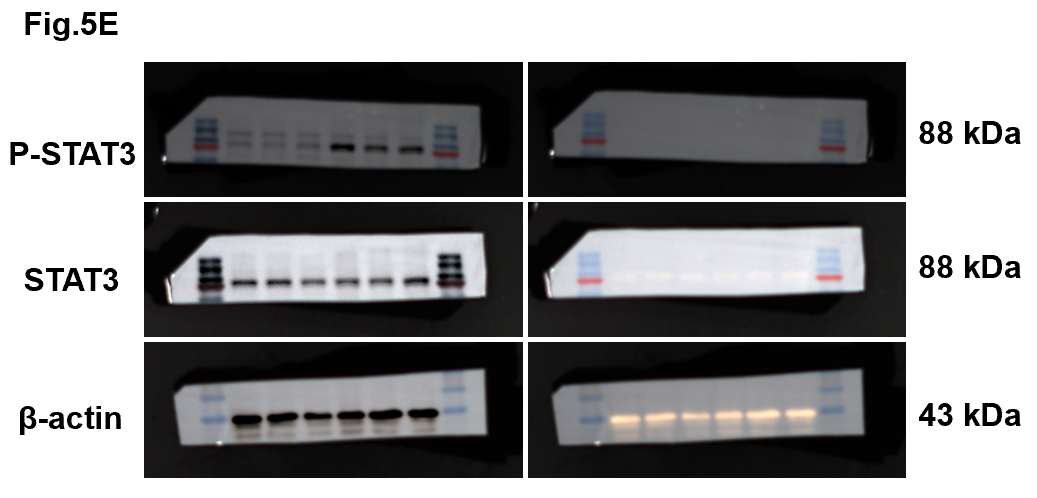


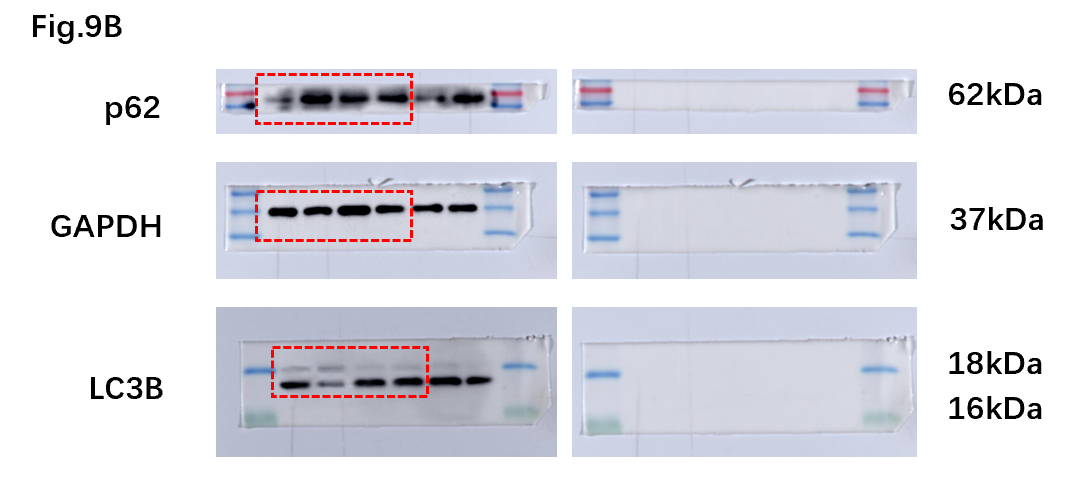

Supplement: Supplementary file 2 — Western blot raw [file 41419_2026_8675_MOESM2_ESM.docx]
